# Identification and characterization of maize microRNAs involved in the very early stage of seed germination

**DOI:** 10.1186/1471-2164-12-154

**Published:** 2011-03-18

**Authors:** Liwen Wang, Huaihua Liu, Detao Li, Huabang Chen

**Affiliations:** 1 State Key Laboratory of Crop Biology, Shandong Agricultural University, Taian, Shandong 271018, PR China

## Abstract

**Background:**

MicroRNAs (miRNAs) are a new class of endogenous small RNAs that play essential regulatory roles in plant growth, development and stress response. Extensive studies of miRNAs have been performed in model plants such as rice, *Arabidopsis thaliana *and other plants. However, the number of miRNAs discovered in maize is relatively low and little is known about miRNAs involved in the very early stage during seed germination.

**Results:**

In this study, a small RNA library from maize seed 24 hours after imbibition was sequenced by the Solexa technology. A total of 11,338,273 reads were obtained. 1,047,447 total reads representing 431 unique sRNAs matched to known maize miRNAs. Further analysis confirmed the authenticity of 115 known miRNAs belonging to 24 miRNA families and the discovery of 167 novel miRNAs in maize. Both the known and the novel miRNAs were confirmed by sequencing of a second small RNA library constructed the same way as the one used in the first sequencing. We also found 10 miRNAs that had not been reported in maize, but had been reported in other plant species. All novel sequences had not been earlier described in other plant species. In addition, seven miRNA* sequences were also obtained. Putative targets for 106 novel miRNAs were successfully predicted. Our results indicated that miRNA-mediated gene expression regulation is present in maize imbibed seed.

**Conclusions:**

This study led to the confirmation of the authenticity of 115 known miRNAs and the discovery of 167 novel miRNAs in maize. Identification of novel miRNAs resulted in significant enrichment of the repertoire of maize miRNAs and provided insights into miRNA regulation of genes expressed in imbibed seed.

## Background

In recent years, the discovery of numerous small RNAs has a great deal of interest in post-transcriptional gene expression regulation during development and other biological processes. Small RNAs (sRNA) include several kinds of short non-coding RNAs, such as microRNA (miRNA), small interfering RNA (siRNA), and Piwi-associated RNA (piRNA), which all regulate gene expression at the post-transcriptional level. The sRNA content of plant cells is surprisingly complex, suggesting an extensive regulatory role for these molecules [1]. The best-characterized class of plant sRNAs is miRNAs [2]. Typically, miRNAs are approximately 22 nucleotide small-RNA sequences that play key roles in many diverse biological processes, including development, viral defense, metabolism and apoptosis [3].

MicroRNAs (miRNAs) are generated from precursor RNA (pre-miRNA) with hairpin structures by DICER-LIKE 1 (DCL1) [4]. DCL1 trims the hairpin structure (pre-miRNA), and then a further cleavage by the same enzyme releases the miRNA/miRNA* duplex [5]. This duplex has a 2-nt 3-overhang at each side and contains a few mismatches [6]. One of the strands of the generated miRNA/miRNA* duplex is incorporated into the RNA-induced silencing complex (RISC). This strand is usually the mature miRNA strand and the miRNA* strand gets degraded, although in some cases the miRNA* strand also accumulates at a lower level [6]. The incorporated mature miRNA guides RISC to mRNAs containing a target site and RISC down-regulates the expression of the mRNA. The 'seed' region located at miRNA nucleotides 2-8 is the most important sequence for interaction with mRNA targets [7]. In plants the target site shows near perfect complementarity to the miRNA sequence, and as a consequence most target mRNAs are cleaved by RISC, although there are examples where the translation of the mRNA is suppressed without a cleavage [8].

MicroRNAs' regulatory role has been exemplified by the critical regulatory behavior of miRNAs at key positions in a variety of pathways, such as root [9], shoot [10], leaf [11], flower development [12] and cell fate [13]. Additionally, they also include responses to phytohormones [14], nutrient [15] and other environmental stresses [16-18]. Furthermore, the targets of several miRNAs are genes that play important roles in stress tolerance, including the gene encoding Cu/Zn SOD [19]. MiR393 targets auxin receptor genes, such as TIR1, AFB2 and AFB3, which lower auxin signals and inhibit the pathogen P. *syringae *[20]. MiRNAs are also induced by pathogens, which suggests miRNAs are involved in plant-microorganism interactions such as symbiosis events with legumes and rhizobia bacteria [21,22]. Increasing evidence demonstrates that miRNAs might provide a novel platform to better understand plant development and resistance to biotic as well as abiotic stresses.

Currently, 14,197 mature miRNAs have been discovered and deposited in the public available miRNA database miRBase (Release 15.0, April 2010) [23]. These miRNAs include 2,566 miRNAs from 37 plant species. The study of small RNAs in maize has been reported but compared with other crops such as rice the number of maize miRNAs identified so far is relatively small [24]. The number of maize miRNAs is even smaller than that of *Arabidopsis *though maize genome size is much larger than that of *Arabidopsis*. To date, there are 170 maize miRNAs, 447 rice miRNAs and 199 *Arabidopsis *miRNAs in miRBase. The identification of a near complete set of small RNAs in any organism is of fundamental importance to understanding small RNA-mediated gene regulations and the diversity of small RNAs. It lays the necessary foundation for unavailing the complex small RNA-mediated regulatory networks. Maize is an obvious choice for high-throughput small RNA sequencing, because of its worldwide agricultural importance, besides being a C4 plant with a sequenced genome.

The most challenging problem in understanding plant miRNAs is to identify more novel miRNAs. Three major approaches have been used for miRNA discovery in plants: forward genetics, bioinformatics prediction as well as direct cloning and sequencing. Only a few miRNAs were identified by forward genetic studies and predicting species-specific miRNAs using bioinformatics method was difficult [25,26]. Thus, direct cloning and sequencing is the most effective method for plant miRNA discovery. Recently, the deep sequencing technology has revolutionized small RNA discovery and more and more miRNAs have been identified. This study leads to the confirmation of the authenticity of 115 known miRNAs and the discovery of 167 novel ones in maize. Identification of novel miRNAs results in significant enrichment of the repertoire of maize miRNAs and provides insights into miRNA regulation of genes expressed in imbibed seed.

## Results and Discussion

### Deep sequencing of maize short RNAs

In order to identify the miRNAs involved in the very early stage of seed germination, a small RNA library from maize seed 24 hours after imbibition was sequenced by the Solexa technology. A total of 11,338,273 reads were obtained. After removing the low quality sequences and adapter sequences, 9,731,557 reads were obtained with 18-30nt in length (Additional file 1). We then further removed sequences that were read only once and 6,870,535 reads remained. Next, all Solexa reads were aligned against the Maize B73 RefGen_v2 (release 5a.57 in May, 2010) using SOAP and 5,791,874 reads were perfectly matched to the maize genome [27], representing 84.3% of the total reads. Around 5.08% of the distinct reads matched noncoding RNAs including rRNAs (2.81%), tRNAs (0.34%), snRNAs (0.06%), siRNAs (1.82%) and snoRNAs (0.05%), which accounted for 16.11% of the total reads (Table 1). All the sequences excluding noncoding RNAs were then regarded as miRNAs for further analysis.

**Table 1:** Summary of read signatures that match various RNAs

Locus class	Distinct signatures	Total signatures
Nonprotein-coding RNAs		
snoRNA	387(0.05%)	6695(0.10%)
snRNA	465(0.06%)	5567(0.08%)
tRNA	2596(0.34%)	195000(2.84%)
rRNA	21675(2.81%)	701447(10.21%)
siRNA	14026(1.82%)	198156(2.88%)
Small RNA matching protein-coding genes		
exon_antisense	17284(2.24%)	116288(1.69%)
exon_sense	30111(3.91%)	265100(3.86%)
intron_antisense	33978(4.41%)	274188(3.99%)
intron_sense	93438(12.12%)	825860(12.02%)
miRNAs		
Known	431(0.06%)	1047447(15.25%)
Novel	1068(0.14%)	49333(0.72%)
Other smallRNAs	555208(72.04%)	3185454(46.36%)
Total	770667	6870535

**Figure 1:**
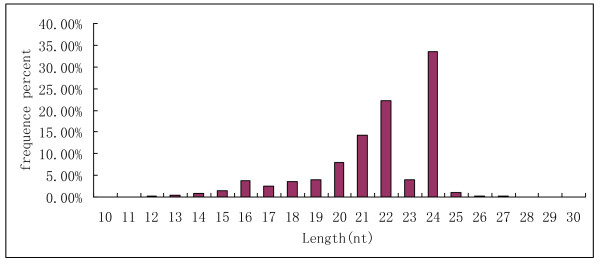
**Length distribution and abundance of the sequence**.

**Figure 2:**
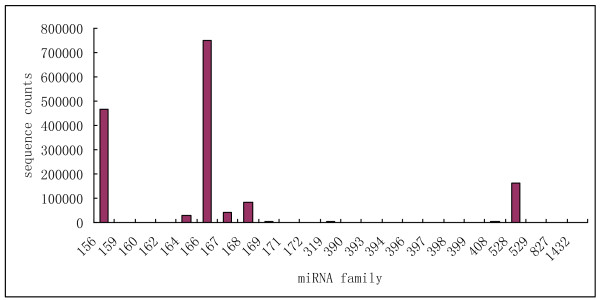
**Abundance of conserved miRNAs sequences in maize small RNA libraries**.

In maize, the size of the small RNAs was not evenly distributed. Among these sequences shown in Figure 1, the number of 24nt sequences was significantly greater than other sequences and accounted for 33.43% of the total reads. This result was consistent with that of *Medicago truncatula *[28], rice [29], peanut [30] and *Arabidopsis *[31]. However, the size distribution differed from wheat and conifer miRNAs obtained through 454 high-throughput sequencing [29,32] and Chinese yew sequences obtained through Solexa technology [33]. In conifer, the fraction of 24nt microRNAs was very small (2.6%) due to the lack of DCL3, the enzyme that matured 24nt RNAs in angiosperms [29,34]. The next larger fractions were the 22nt (22.20%), 21nt (14.29%) and 20nt (7.87%) fractions, representing the typical length of plant mature miRNAs. Intriguingly, the fraction of 23nt miRNAs was very small (3.5%) compared to those of 21, 22 and 24nt fractions. The same phenomenon was also observed in peanut [30], cotton [35] and *Medicago truncatula *[28]. As shown in figure 2, the abundance of miRNAs in our dataset varied drastically. Some were sequenced only a few times, whereas others were present thousands of times, indicating many physiological and biochemical processes are being carried out and maize seed contains a large and diverse small RNA population at the very early stage of seed germination.

### Identification of conserved maize miRNAs

**Figure 3:**
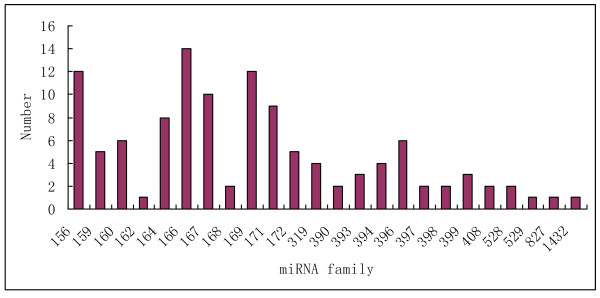
**Number of detected family members per miRNA family**. Candidate miRNA families were taken together and grouped by their miRBase numerical identifiers.

Conserved miRNA families are found in many plant species and have important functions in plant development. To identify conserved miRNAs in our dataset, all small RNA sequences were Blastn searched against the known maize mature miRNAs and their precursors in the miRNA database miRBase. There are currently 28 families containing 170 known miRNAs in miRBase. Blastn searches showed that 1,047,447 total reads representing 431 unique sRNAs matched to known maize miRNAs. Further analysis identified a total of 115 conserved miRNAs that belong to 24 miRNA families (Additional file 2). The identified miRNA families have been shown conserved in a variety of plant species. For example, miR156/157, miR159/319, miR166, miR169, and miR394 have been found in 51, 45, 41, 40 and 40 plant species, respectively [36-38]. Maize miRNA families displayed significantly varied abundance from each other. This varied abundance of the miRNA families suggested that miRNA genes would be differentially transcribed at this very early stage of seed germination. For example, the majority of maize miRNAs were only sequenced less than 1,000 times, and some rare miRNAs were detected less than 10 times, whereas zma-miR167a, zma-miR166a, and zma-miR156a were detected 27,634, 300,503 and 374,492 times respectively (Additional file 2). The abundance of zma-miR172 was extremely low compared to that of zma-miR156 in our dataset, which was consistent with previous finding that these two miRNAs are conversely regulated. In comparison to other plant species, tae-miR169b in wheat and osa-miR169 in rice are the most frequently sequenced miRNAs while miR156 in rice and wheat exhibits low abundance [32]. This may suggest a species-specific expression profile for miRNAs. MiR156a was also found to be highly expressed in *Medicago truncatula *[39]. In *Arabidopsis*, miR156a, located on chromosome 2, targets 10 mRNAs that code for the squamosa promoter-binding protein (SBP) box, which is involved in leaf morphogenesis [39,40]. However, mechanisms causing the differential expression profile of a same miRNA in different plant species is unknown. Diversity of maize miRNAs also could be found in the aspect of the amount of members they contained (figure 3). The largest miRNA family size identified was miR166 that consisted of 14 members and miR156, miR169 and miR167 possessed 12, 12 and 10 members, respectively; whereas other miRNA families such as miR162, miR529, miR827 and miR1432 had only one member detected in this period. The size of miRNA families may be indicative of their function. Different family members also displayed drastically different expression levels (Additional file 2). For example, the abundance of miR156 family varied from 261 reads (zma-miR156j) to 409,637 reads (zma-miR156d) in the deep sequencing. This was also the case for some other miRNA families, such as zma-miR164 (from 14 read to 25,253 reads) and zma-miR166 (from 931 reads to 300,478 reads). Two members, zma-miR528a and zma-miR528b in zma-miR528 family, however, their expression levels were similar and were detected 147,619 and 158,200 times, respectively. The existence of a dominant member in a miRNA family may suggest that the regulatory role of this family was performed by the dominant member at the developmental time when the samples were collected for RNA extraction. Abundance comparisons of different members in one miRNA family may provide valuable information on the role that miRNAs play in that plant specific developmental stage. Four known miRNA families, miR395, miR482, miR2118 and miR2275 were not successfully detected in our datasets suggesting that miRNAs expression maybe developmental and/or tissue-specific. After our dataset was Blastn searched against known maize mature miRNAs, the same dataset was used to compare with 2392 known miRNAs from other diverse plant species. We found 10 additional conserved miRNAs that have not been reported in maize (Additional file 3). The 'seed' region, located at miRNA nucleotides 2-8, is the most important sequence for interaction with mRNA targets [7]. The seed regions of the newly identified maize miRNAs t0002967, t0511822, t0207061, t0448353 and t0053880 were identical to those of ctr-miR171 and ctr-miR166, respectively, indicating that they may share the same targets.

### Identification of novel maize miRNAs

Although the characteristic hairpin structure of miRNA precursor could be used to predict novel miRNA, it is very challenging to define novel miRNAs. We developed a prediction software Mireap to predict novel miRNA by exploring the secondary structure, the Dicer cleavage site and the minimum free energy of the unannotated small RNA tags which could be mapped to the maize genome. A small RNA is considered as a potential miRNA candidate only if it meets all of the following strict criterias: 1) the sequence could fold into an appropriate stemloop hairpin secondary structure, 2) the small RNA sits in one arm of the hairpin structure, 3) no more than 6 mismatches between the predicted mature miRNA sequence and its opposite miRNA* sequence in the secondary structure, 4) no loop or break in the miRNA or miRNA* sequences, and 5) predicted secondary structure has higher minimal folding free energy index and negative minimal folding free energy. 1068 sequences were obtained based on the above criteria. Although forming specific hairpin stem loop structures is one of the most important characteristics of pre-miRNAs, it is not unique to pre-miRNAs; lots of other coding or non-coding RNAs, such as rRNAs, tRNAs and mRNAs, also have the similar hairpin structures [41]. Several studies observed that miRNA precursors have low folding free energy, and considered that low free energy is one important characteristic of miRNAs [42]. However, minimal folding free energy depends on the lengths of RNAs [43] and the length of miRNA precursors significantly varies, for example, the lengths of plant miRNA precursors range from 60 to more than 400 nucleotides [41]. To avoid the effect of using minimal folding free energy as a criterion to identify genuine miRNAs, the length of RNAs must be considered. To better distinguish miRNAs from other RNAs, Zhang et al. [41] combined several parameters to form a new criterion called minimal folding free energy index (MFEI) [41]. Pre-miRNAs have high minimal folding free energy index (MFEI) [41]. They found that the average MFEI of miRNA precursors is 0.97 in previously known plant pre-miRNAs, and this value is significantly higher than that for tRNAs (0.64), rRNAs (0.59), and mRNAs (0.62-0.66). More importantly, more than 90% of miRNA precursors have an MFEI greater than 0.85, and no other RNAs have MFEI higher than 0.85. This suggests that MFEI is useful to distinguish miRNAs from other non-coding and coding RNAs. Their results suggest that RNA sequences with MFEI larger than 0.85 are most likely to be miRNAs [41]. This finding provids a more precision criterion to predict miRNAs using computational and/or experimental approaches. Out of the 1068 miRNA candidates, 386 had MFEI greater than 0.85.

In order to make certain that the 386 miRNA candidates we identified are true miRNAs, we constructed and sequenced a second small RNA library from the same tissue. Following the same analysis approach as that used for the first library, 362 sequences with MFEI greater than 0.85 were identified. By comparing these 386 and 362 distinct reads, we found that 167 of them were identical between the two small RNA libraries in terms of their precursor sequences, mature miRNA sequences, and their chromosomal locations. Further more, no significant differences of their expression profiles existed between the two experiments. We believed that these 167 sequences were most likely true novel miRNAs. The stringent criteria used to predict novel miRNAs could potentially reduce false positive rates at the cost of missing authentic miRNAs.

**Figure 4:**
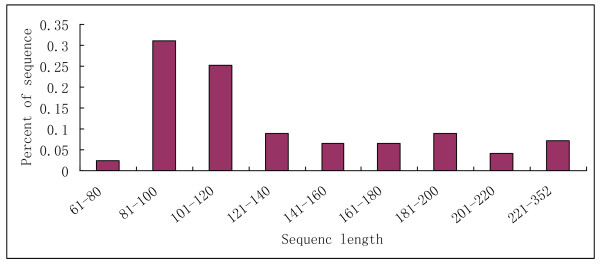
**Size distribution of novel miRNAs**.

The 167 novel miRNAs can be classified into 77 families (Additional file 4) and their pre-miRNAs, secondary structures, and chromosomal locations were listed in Additional file 5. These novel miRNA precursors had negative folding free energies (20.8-210 kcal mol^-1 ^with an average of about -68.8 kcal mol^-1^) according to Mfold3.2 (Additional file 4) [44]; this was similar to the computational values of *Arabidopsis thaliana *miRNA precursors (-57 kcal mol^-1^) and much lower than folding free energies of tRNA (-27.5 kcal mol^-1^) or rRNA (-33 kcal mol^-1^) [42]. Previous study indicated that animal miRNA precursors typically have 70-80 nucleotides, but plant miRNA precursors are more diverse in structure and size. They vary in size from 60 to 509 nucleotides, with an average of 144.6 ± 56.9 (n = 513); most (73.5%) of the detected miRNAs have 81-160 nucleotides. Only 1.6% of plant miRNAs are less than 81 nucleotides in length, a stark contrast to animal miRNAs [45]. In our research as shown in figure 4, the foldback precursors of 167 novel miRNAs were about 67-356 nucleotides in length, and about 71.9% with 81-160 nucleotides. The novel pre-miRNAs were also evaluated for their A+U content, which ranged from 34% to 69.01% (Additional file 4), in agreement with previous studies. We also looked for sequenced miRNA* sequences, only seven complementary sequences were found in our combined data sets (Additional file 4). Most miRNA* shows weak expression (sequencing frequency <10) and their expression levels are much lower than their corresponding miRNAs, consistent with the idea that miRNA* strands are degraded rapidly during the biogenesis of mature miRNAs [46]. It may also be the fact that the expression levels of the majority of the novel miRNAs identified were low (the majority of them were sequenced less than 50 times).

### Target prediction of maize miRNAs

As dry seeds imbibe water, the resumption of energy metabolism and cellular repair occur. Later, events such as the activation of genes encoding enzymes involved starch degradation and protein and DNA/RNA synthesis play critical roles in the decision as to whether a seed would germinate or not. The shift from the seed development/maturation mode to the germination mode is a critical change in the developmental program of seed. Regulation of transcription factors targeted by miRNAs is involved at this critical stage in plant development [47]. Our target prediction criteria and methods were also stringent, but still allowed us to capture most miRNA targets that are conserved across several plant species, including *Arabidopsis *[48], poplar [17], rice [49], wheat [50], soybean [51], mustard [52] and grape [53]. The majority of conserved miRNA targets are various transcriptional factors including SBP, MYB, ARF, NAM, CBF, TCP and GRF that are known to regulate plant development. Other conserved miRNA targets includes F-box protein (miRNA393, miRNA394), ATP sulfurylase (miRNA395), CCHC type zinc finger protein (miRNA482), NAD(P)-binding protein (miRNA827), and Poly(ADP-ribose) polymerase (miRNA1432), all of them are known to play roles in the expression control of genes involved in regulation of metabolic processes. In our datasets, miRNA166 showed the highest abundance followed by miRNA156 and miRNA528, respectively, during the very early stage of seed germination. Previous studies indicated that MiRNA166 targets HD-ZIP transcription factors that are involved in plant leaf morphogenesis. HD-ZIP proteins also regulates vascular development as well as lateral organ polarity and meristem formation. ATHB15, a member of the HD-ZIP family, is predominantly expressed in vascular tissues, suggesting that it may play some roles in plant vascular development [54,55]. Overexpression of miR166a results in decreasing ATHB15 mRNA levels and causes accelerated vascular cell differentiation from cambial/procambial cells and consequently produces an altered vascular system with expanded xylem tissue and an interfascicular region [55]. This regulation mechanism may exist in all vascular plant species [55,56]. MiRNA156 has been shown to be involved in floral development and phase change by targeting members of squamosa promoter binding protein like (SPL) plant-specific transcription factors. The SPL family has 16 members; some (such as SPL3) are involved in floral transition and regulating plant flowering [57]. Recent results indicated that overexpression of miR156 affects phase transition from vegetative growth to reproductive growth, including the quickly initiation of rosette leaves, a severe decrease in apical dominance, and a moderate delay in flowering [58]. MiRNA528 targets copper proteins, cupredoxin, multicopper oxidase and laccase genes and thus might play a critical role in regulating physiological processes and stress responses. Not only the miRNA166 and miRNA156 families were abundant during this stage of seed germination, but also they had more family members than other miRNA families, suggesting the importance of these two miRNA families at this very early stage of seed germination. In *Arobidopsis*, MiRNA159 has been shown to be involved in the regulation of seed dormancy and germination by targeting *MYB33 *and *MYB101*, two positive regulators of ABA responses during germination. ABA is a key regulator of seed maturation and dormancy [59]. Many ABA signal transduction proteins are involved in seed development and germination [59-62]. The sensitivity of seeds to ABA that is vital to the termination of seed maturation program, an essential change to increase the competence of seeds for germination, is regulated by conserved miRNA160. Since there is no dormancy in maize seed, the abundance of both miRNA159 and miRNA160 is extremely low compared to that of miRNA166 and miRNA156 families in our datasets. *AUXIN RESPONSIVE FACTORs *(ARFs) are a class of targets of miRNA160 families. ARFs are important components of auxin signal transduction [63]. Therefore, there is cross-talk between ABA and auxin in imbibed mature seeds. Studies has indicated that ABA-responsive genes that are typical of seed maturation stages and have ABA response elements (ABREs) in their promoter regions are specifically up-regulated in the miRNA-resistant *mARF10 *seeds. The down-regulation of a component important for auxin signal transduction by miRNA may be a regulatory step to decrease ABA sensitivity in mature seeds and to switch to the germination mode. The mechanisms involved in ABA-auxin cross-talk during seed germination are unknown.

To better understand the functions of the newly identified novel miRNAs in maize, putative targets of the 167 novel miRNAs were predicted. The target genes for 106 novel miRNAs were successfully predicted (Additional file 6). Analysis and annotation of the predicted target genes showed that they were with diverse functions, ranging from genes encoding transcription factors involved in transcription regulation to genes encoding enzymes involved in metabolism, genes regulating transport, genes encoding various kinases, genes regulating oxidative reduction and genes encoding isomarase and helicase (Additional file 6). When dry seeds absorb water, many cellular processes resume. Isomarase and helicase are important enzymes for DNA replication, transcription, translation, recombination, DNA repair, ribosome biogenesis. Most miRNA families have multiple target sites, suggesting that these miRNAs are functionally divergent. With 61 newly identified miRNAs, we failed to discover any targets for them in Maize. This could have resulted from incomplete coverage of the mRNA in the database. It is likely that a number of mRNAs could not be identified because they are poorly expressed or highly unstable, or because their expression is restricted to times and locations such that isolation of sufficient amounts of RNA for cloning is impractical or has not been done yet. Further analysis for their targets is needed and would help us gain insight into the roles these newly identified miRNAs play during maize seed imbibition.

## Conclusion

We had sequenced two independent small RNA libraries from maize imbibed seed. Our data confirmed the authenticity of 115 known miRNAs in maize. We found 10 miRNAs that had not been reported in maize, but had been reported in other plant species. We also found 167 novel miRNAs that had not been reported elsewhere. Putative targets for 106 novel miRNAs were predicted. Dry seeds imbibe water and re-initiate active physiology. An important decision as to whether a seed would germinate or not is made following the reactivation events during imbibition. Regulation of genes targeted by miRNAs is involved at this critical stage in plant development. Identification of novel miRNAs resulted in significant enrichment of the repertoire of maize miRNAs and provided insights into miRNA regulation of genes expressed in imbibed seed.

## Methods

### RNA isolation and cloning of maize small RNAs

Maize (*Zea mays*) inbred line 87-1 was used in this study. Seeds were sterilized, wrapped in paper towels and incubated at 25°C for 24 hours. Embryos were then cut out and used for RNA extraction. Briefly, total RNA was isolated using Trizol kit (Invitrogen, USA). Small RNAs were enriched by poly-ethylene glycol precipitation, separated on 15% denaturing PAGE, and visualized by SYBR-gold staining. Small RNAs of 16-28nt were gel-purified. Small RNAs were ligated to a 5'adaptor and a 3'adaptor sequentially, reverse-transcription polymerase chain reaction (RT-PCR) amplified, and used for sequencing directly [64]. Sequencing was performed on a Solexa machine (Beijing Genomics Institute, China).

### Identification of conserved and novel miRNAs

The raw sequences were processed using PHRED and CROSS MATCH programs as previously reported [18,65]. After removing the vector sequences, trimmed sequences longer than 18 nt were used for further analyses. First, rRNA, tRNA, snRNA, and snoRNA, as well as those containing the polyA tail, were removed from the small RNA sequences and the remaining sequences were compared against maize ncRNAs deposited in the NCBI Genbank database and Rfam database. Then, the unique small RNA sequences were used to do a Blastn search against the miRNA database, miRBase 15.0, in order to identify conserved miRNAs in maize. Only those small RNAs whose mature and precursor sequences perfectly matched known maize miRNAs in miRBase 15.0 were considered to be conserved miRNAs.

To discover potential novel miRNA precursor sequences in our dataset, we used the identified mature miRNA sequences to do Blastn searches against maize genomic sequence. Sequences that met previously described criteria were then considered to be miRNA precursors [66]. Specifically, dominant, mature sequences residing in the stem region of the stem-loop structure and ranging between 20-22 nt with a maximum free-folding energy of -20 kcal mol^-1^were considered. A maximum of six unpaired nucleotides between the miRNA and miRNA* was allowed. The distance between the miRNA and miRNA* ranged between 5 and 240-nt. The selected sequences were then folded into a secondary structure using an RNA-folding program mFold3.2. If a perfect stem-loop structure was formed, the small RNA sequence was sit at one arm of the stem as well as other criteria were followed, this small RNA was consisted as one potential novel maize miRNA candidate. Previous study indicated that more than 90% of miRNA precursors had an MFEI greater than 0.85, and no other RNAs had MFEI higher than 0.85 (MFEI = [(MFE/length of the RNA sequence) × 100]/(G+C)% [67]). This suggested that MFEI is useful to distinguish miRNAs from other non-coding and coding sRNAs. The MFEI was calculated and potential novel maize miRNA candidates were further screened.

### Target gene prediction

In order to predict the target genes of novel miRNAs, we used the Mireap method for target prediction [68,69]. Briefly, the criteria were as follows: 1) No more than four mismatches between sRNA & target (G-U bases count as 0.5 mismatches), 2) No more than two adjacent mismatches in the miRNA/target duplex, 3) No adjacent mismatches in positions 2-12 of the miRNA/target duplex (5' of miRNA), 4) No mismatches in positions 10-11 of miRNA/target duplex, 5) No more than 2.5 mismatches in positions 1-12 of the of the miRNA/target duplex (5' of miRNA), and 6) Minimum free energy (MFE) of the miRNA/target duplex should be > = 75% of the MFE of the miRNA bound to it's perfect complement. Target mRNA sequences were predicted follow the criteria for the identified novel miRNAs (see Additional file 4). More strictly, at most three mismatches between miRNA sequences and potential mRNA targets were allowed in this study and grouped by the biological function of the proteins they encode for, as described by UniProt (http://www.uniprot.org/).

### Analysis of sequencing data

Raw sequence reads were produced by the Illumina 1G Genome Analyzer at BGI-Shenzhen, China and processed into clean full length reads by the BGI small RNA pipeline. During this procedure all low quality reads, including 3' adapter reads and 5' adapter contaminants were removed. The remaining high quality sequences were trimmed of their adapter sequences and sequences larger than 30nt and smaller than 18nt were discarded. All high quality sequences, even those with only a single unique read, were considered as significant and further analyzed. Unique small RNA sequences were mapped to maize genome (B73 RefGen_v2 (release 5a.57 in May, 2010)) reference sequences by SOAP [70]. Small RNAs derived from rRNAs, tRNAs, snRNAs and snoRNAs deposited at the Rfam and NCBI GenBank databases http://www.ncbi.nlm.nih.gov/Ftp/ were identified by NCBI blast. In order to determine conserved miRNAs, unique sequences were aligned with known maize miRNAs from miRBase (Release15.0, Apil, 2010) with a maximum of two mismatches, where gaps count as mismatches. Potential novel miRNAs were identified by folding the flanking genome sequence of unique small RNAs using MIREAP (https://sourceforge.net/projects/mireap/), followed by the prediction of the secondary structure by mFold 3.2 [71]. The essential criteria were used for selecting the miRNA candidates, e.g. sequences of miRNA precursors can fold into a hairpin secondary structure that contains the ~21nt mature miRNA sequence from one arm and miRNA*derived from the opposite arm, both of which form a duplex with two nucleotide, 3' overhangs [72]. The filtered small RNA sequencing data were deposited in the National Center for Biotechnology Information Gene Expression Omnibus (http://www.ncbi.nlm.nih.gov/projects/geo/) under accession number GSE27664.

For prediction of miRNA targets, the procedure and criteria were followed as described previously [73,74]. More strictly, at most three mismatches between miRNA sequences and potential mRNA targets were allowed in this study. The biological function of the predicted targets was retrieved from the UniProt.

## Authors' contributions

LW participated in the data analysis and drafted the manuscript. HL participated in the design of the study and performed the miRNA targets prediction. DL carried out RNA extraction and sequence data analysis. HC conceived of the study, participated in its design and coordination, revised the manuscript and gave final approval of the version to be published. All authors read and approved the final manuscript.

## Supplementary Material

Additional file 1 **Summary of small RNA sequencing date**. Click here for file

Additional file 2 **Conserved miRNAs in maize**. These miRNAs are conserved in maize and have been reported in miRBase. "+"present in our dataset, "-"absent in our dataset. *L *length. **: miRNA sequences of maize were identical to those in other species; *: maize miRNA sequences were conserved in other species but have variations in some nucleotide positions. Click here for file

Additional file 3 **Ten conserved maize miRNAs identified in this study**. Click here for file

Additional file 4 **Novel miRNAs identified in this study**. Click here for file

Additional file 5 **Secondary structures of novel miRNA in maize**. Red colored letter: mature miRNA sequence; blue colored letter: miRNA* sequence. Click here for file

Additional file 6 **Predicted targets for novel miRNAs**. MiRNAs targeting the same gene and site are grouped together. The first listed miRNA has the least mismatches. "Mm" stands for the total amount of mismatches between the first mentioned miRNA and the predicted target. "Alignment" visually represents miRNA/mRNA complementary base-pairs and mismatches for the first listed miRNA, with vertical bars and spaces as Watson-Crick base-pairs and mismatches, respectively (G:U wobbles count as mismatches). Click here for file
