# Supplementary material for: Identification and characterization of maize microRNAs involved in the very early stage of seed germination
Source: BMC Genomics. 2011 Mar 18;12:154. doi: 10.1186/1471-2164-12-154 (PMC3066126; doi:10.1186/1471-2164-12-154)
Supplement: Additional file 1 — Summary of small RNA sequencing date. [file 1471-2164-12-154-S1.DOC]

## Additional file 1 Summary of small RNA sequencing date

| Type | count | % |
| --- | --- | --- |
| total_read | 11328237 |  |
| high_quality | 10830740 | 100% |
| adaptor3_null | 8995 | 0.08% |
| insert_null | 2687 | 0.02% |
| adaptor5_contaminants | 89477 | 0.83% |
| small_than_18nt | 997615 | 9.21% |
| polyA | 409 | 0.00% |
| clean_reads | 9731557 | 89.85% |
