# Supplementary material for: Identification and characterization of maize microRNAs involved in the very early stage of seed germination
Source: BMC Genomics. 2011 Mar 18;12:154. doi: 10.1186/1471-2164-12-154 (PMC3066126; doi:10.1186/1471-2164-12-154)
Supplement: Additional file 3 — Ten conserved maize miRNAs identified in this study. [file 1471-2164-12-154-S3.DOC]

**Additional file 3 Ten conserved maize miRNAs identified in this study***

| Names | Sequences | Target gene | Target protein |
| --- | --- | --- | --- |
| t0002967 | UUGAGCCGCGUCAAUAUCUCC | GRMZM2G545615 | unknown |
| ctr-miR171 | UUGAGCCGCGUCAAUAUCUCC | No[1] |  |
|  |  |  |  |
| t0004555 | UGAUAGAAGAGAGUGAGCAC | GRMZM2G3-9--6 | hypothetical protein |
| ath-miR156a | UGACAGAAGAGAGUGAGCAC | At5g43270 | SBP[2] |
|  |  |  |  |
| t0053880 | UCGGACCAGGCUUCAUUCCCCG | GRMZM2G-96372 | hypothetical protein |
| ctr-miR166 | UCGGACCAGGCUUCAUUCCCCC | 5509 (3) | HD-ZIP protein |
|  |  |  |  |
| t0062267 | UUGAUAGAAGAGAGUGAGCAC | GRMZM2G551565 | unknown |
| ahy-miR156c | UUGACAGAAGAGAGAGAGCAC | ES767441 | Squamosa promoter-binding protein[3] |
|  |  |  |  |
| t0076933 | AGACUUAGGAACGGAGGGAGU | No |  |
| osa-miR1436 | ACAUUAUGGGACGGAGGGAGU | Os03g26190(3); | hypothetical proteins[4] |
|  |  |  |  |
| t0137133 | UGAAACUGUCACAGCAUGAUCUA | No |  |
| ath-miR167a | UGAAGCUGCCAG - - CAUGAUCUA | At5g37020 | ARF[2] |
|  |  |  |  |
| t0207061 | UUGAGCCGCGUCAAUAUCUC - |  |  |
| ctr-miR171 | UUGAGCCGCGUCAAUAUCUCC |  |  |
|  |  |  |  |
| t0448353 | - CGGACCAGGCUUCAUUCCCCG |  |  |
| ctr-miR166 | UCGGACCAGGCUUCAUUCCCCC |  |  |
|  |  |  |  |
| t0511822 | - - GAGCCGCGUCAAUAUCUCC |  |  |
| ctr-miR171 | UUGAGCCGCGUCAAUAUCUCC |  |  |
|  |  |  |  |
| t0724812 | UCGAAAGGGAUUGGAGGGGAU | No |  |
| gma-miR2119 | UCAAAGGGAGUUGUAGGGGAA | 171648484 | Peptidyl-prolyl cis-trans isomerase[5] |

***Maize conserved miRNAs were pairwise listed with maize miRNA on top. Maize conserved miRNAs were homologous/identical to miRNAs in other plant species.**

1. Song C, Fang G,Li X, Liu H, Chao CT: **Identification and characterization of 27 conserved microRNAs in citrus.** *Planta* 2009, **230**:671–685.

2. Kasschau KD, Xie G, Allen E, Llave C, Chapman EJ, Krizan KA, Carrington JC: **P1/HC-Pro, a Viral Suppressor of RNA Silencing, Interferes with Arabidopsis Development and miRNA Function.** *Developmental Cell* 2003, **4**:205–217.

3. Zhao CZ,Xiao H, Frazier TP, Yao YY, Bi YP, Li AQ, Li MG, Li CS, Zhang BH, Wang XJ:**Deep sequencing identifies novel and conserved microRNAs in peanuts (*Arachis hypogaea L*.).** *BMC Plant Biology* 2010, 10:3.

4. Sunkar R, Zhou X, Zheng Y, Zhang W, Zhu J: **Identification of novel and candidate miRNAs in rice by high throughput sequencing.** *BMC Plant Biology* 2008, 8:25.

5. Arenas-Huertero C, Perez B, Rabanal F, Blanco-Melo D, Rosa CD, Estrada-Navarrete G, Sanchez F, Covarrubias AA, Reyes JL: **Conserved and novel miRNAs in the legume Phaseolus vulgaris in response to stress.** *Plant Mol Biol* 2009, **70**:385–401
