# Supplementary material for: Identification and characterization of maize microRNAs involved in the very early stage of seed germination
Source: BMC Genomics. 2011 Mar 18;12:154. doi: 10.1186/1471-2164-12-154 (PMC3066126; doi:10.1186/1471-2164-12-154)
Supplement: Additional file 5 — Secondary structures of novel miRNA in maize. Red colored letter: mature miRNA sequence; blue colored letter: miRNA* sequence. [file 1471-2164-12-154-S5.DOC]

**Additional file 5 Secondary structures of novel miRNA in maize.**

**(**Red colored letter: mature miRNA sequence; blue colored letter: miRNA* sequence.)

**zma-miRn1a AGGAUGCAUCGUUGGGUCCGG**

**- C g a ACC---| C A**

**CCA AAAAUCg agg gauuggaggggcuaaAAU CU AUUCA U**

**GGU UUUUGGC UCC CUAAUCUCCCCGAUUUUA GA UAAGU U**

**A - A C AGAAAA^ A U**

**zma-miRn1b GGAGGAGAUUGGAGGGGCUAA**

**U AAU-| a A A**

**CCA ACCggagg gauuggaggggcuaaAAUCCCCUUC UAUUCA U**

**GGU UGGCCUCC CUAACCUCCCCGAUUUUAGGGGAGG AUAAGU U**

**C GUUU^ C A U**

**zma-miRn1c GGAGGAGAUUGGAGGGGCUAA**

**-| UAAU a u C A**

**CAAGGG ggagg gauuggaggggc aaAAUUC CUUGCUAUUCA A**

**GUUCCC UCUCC CUAACCUCCCCG UUUUAGG GAACGAUAAGU U**

**C^ UU-- C U A U**

**zma-miRn1d GGAGGAGAUUGGAGGGGCUAA**

**- AA A - --| A**

**CCC GAACCggagg gauuggaggggcuaaAAUC CC CUUAUUCA A**

**GGG UUUGGCUUCC CUAACCUCCCCGAUUUUAG GG GAAUAAGU A**

**C CC C A AA^ C**

**zma-miRn2 AGUGGAUUAGAGGGGCUAAAA**

**-| C a u a a c CC C A**

**CAAAACC GA g gg uu gagggg uaaaaU CCUUCU AUUCA A**

**GUUUUGG CU C CC AA CUCCCC AUUUUA GGAGGA UAAGU A**

**U^ C - U A C U AA A U**

**zma-miRn3 AUGGAGUGGUUUGAGGAGGCU**

**- -| u ug a c A AG A**

**GAG AAGGGGAa ggag guuugagg gg uA AAUCUCUC UAUUCA A**

**CUC UUCCUCUU CCUC CAAACUCC CC AU UUAGGGAG AUAAGU A**

**U G^ U CU C A C CG U**

**zma-miRn4a GGUGAACCACCGGACAUCGCAC**

**ACAGUGUACA C- CA-| GCA**

**GUGC UGUCCGGUG CACCGGACAGC C**

**cacg acaggccac guggCCUGUCG A**

**GGUAAAAGGA cu caa^ AUG**

**80 70 60 50**

**zma-miRn4b GGUGAACCACCGGACAUCGCAC**

**ACAG ACA C- CA-| GCA**

**UCU GUGC UGUCCGGUG CACCGGACAGC C**

**AGA cacg acaggccac guggCCUGUCG A**

**GGAA GGA cu caa^ AUG**

**zma-miRn4c GGUGAACCACCGGACAUCGCAC**

**ACAG ACA C- ACA-| GCA**

**UCU GUGC UGUCCGGU CACCGGACAGC C**

**AGA cacg acaggcca guggCCUGUCG A**

**AGAA GGA cu ccaa^ AUG**

**zma-miRn4d GGUGAACCACCGGACAUCGCAC**

**A| GAUAG ACA - CA- GCA**

**CCC UCU GUGC AUGUCCGGUG CACCGGACAGC C**

**GGG GGA Cacg uacaggccac guggCCUGUCG A**

**-^ AAGA- --- c caa AUA**

**zma-miRn4e GGUGAACCACCGGACAUCGCAC**

**ACAG ACA C- CA-| GCA**

**UCU GUGC UGUCCGGUG CACCGGACAGU C**

**AGA cacg acaggccac guggCCUGUCG A**

**AGGG GGA cu caa^ AUG**

**zma-miRn4f GGUGAACCACCGGACAUCGCAC**

**A G A C- CA-| AU AC**

**CA UCUAC GUGC UGUCCGGUG CACCGGACA GC A**

**GU AGAUG cacg acaggccac gugGCCUGU CG G**

**G G A cu caa^ -- AU**

**zma-miRn4g GGUGAACCACCGGACAUCGCAC**

**ACAGUCUACA C- CA-| GCA**

**GUGC UGUCCGGUG CACCGGACAGC C**

**cacg acaggccac guggCCUGUCG A**

**GACAAAAGGA cu caa^ AUG**

**zma-miRn5 UUUUUUGUCUGGAGAUAUUA**

**G A - .-GCA| UG GAA UC**

**AAGUUAUUUAUGAUAUCUCCAGAU AAAAAAGGCAG UUGGUGCAUGUGC UC GG GGG \**

**UUCGAUAAAUauuauagaggucug uuuuuuCCGUC GACCACGUACACG AG CC CCC A**

**- - A \ ---^ GU AA- AG**

**zma-miRn6a AGAGGGGAUUGGAGGGGCUAA**

**-| AUa g - A**

**CAAGGGAA gaggggauuggag ggcuaaAAUC CCU G**

**GUUCCCUU CUCCCCUAACCUC CCGAUUUUAG GGA C**

**G^ AC- G U A**

**zma-miRn6b AGAGGGGAUUGGAGGGGCUAA**

**U A - g CU - --| A**

**CUA AAA CUagaggggauugga gggcuaaAAU CC UC AUUCA A**

**GGU UUU GGUUUCCUCUAACCU CCCGAUUUUA GG AG UAAGU A**

**C A A G AG A AA^ U**

**zma-miRn6c AGAGGGGAUUGGAGGGGCUAA**

**-| UC a C C A**

**CC AAACUC gaggggauuggaggggcuaaAAU UCCU CUUAUUCA U**

**GG UUUGGG CUCCCCUAACUUCUCCGAUUUUA AGGA GAAUAAGU U**

**A^ U- C - A U**

**zma-miRn6e AGAGGGGAUUGGAGGGGCUAA**

**- - Ua -| c CA**

**GUCAC AAAC gaggggauugga gggg uaaAAUCCCUUCCUUAUU A**

**CGGUG UUUG CUCCCCUAACCU CCCC AUUUUAGGGGAGGAAUAA A**

**U U GC A^ - AA**

**zma-miRn7a AAACCCGGUUUUGGACGGUUU**

**C C A C CCU C - -- .-CCCCCGAACU C-- C--| A**

**GGCCCAU CAAAACC UCCAAAACCGGGUUUGCUCAAA UGGAUG ACGUGGCGUGCUA GUUGGAGU UACU UGAA UGGCAUG UGUCA UUGG U**

**CCGGGUA GUuuugg agguuuuggcccaaaCGAGUUU GCCUAC UGCGCCGCACGGU CAACCUCG AUGA ACUU ACCGUGU ACGGU AGCC C**

**- C c A AAC A U UU \ ---------- CCU UCA^ C**

**zma-miRn7b AAACCCGGUUUUGGACGGUUU**

**U A U---- GC A A AUG .-AAAGUAAC -|U CA G**

**GG UCC AAACCGUCCAAAACCGGGUUU UCAAACC GAUGUC AC GCGUGUCACGUU UUGG G CUC AAGUGGCAU \**

**CC GGG uuuggcagguuuuggcccaaa AGUUUGG CUACGG UG CGCACGGUGCAA AGCC C GAG UUCACCGUA C**

**- - CACGU GA C C AAA \ -------- U^U -- U**

**zma-miRn7c AAACCCGGUUUUGGACGGUUU**

**A C C UUU C CAC .-AAUGAAAG AACCUAA AC**

**AAACCGUCCAAAACCGGGUUU UUCAAACCGGAUG CGAC GCGUG CACGUUGGAG ACU UUCGAG GUG A**

**uuuggcagguuuuggcccaaa GAGUUUGGUCUAC GUUG CGCAC GUGCAAUUUC UGA AGGCUU UAC C**

**A C A UAC A AU- \ -------- AAAUCG- AA**

**zma-miRn8a GGAGGGAAUUGGAGGGGCUA**

**-| UC a g CC A**

**CC AAACCUggaggg auu gaggggcuaAAAUCCCCU UUAUUCA U**

**GG UUUGGGCUUCCC UAA UUCCCCGAUUUUAGGGGA AAUAAGU U**

**A^ U- C A AA U**

**zma-miRn8b GGAGGGAAUUGGAGGGGCUA**

**UC U-| C a g A U U A**

**CAAA AC ggaggg auuggaggg cuaAA UCCCUUCC UAU CA A**

**GUUU UG CCUCUC UAACCUCCC GAUUU AGGGGAGG AUA GU A**

**CU UU^ A C A A U U U**

**zma-miRn8c GGAGGGAAUUGGAGGGGCUA**

**C| AC g C A**

**UAAAAU Uggagggaauuggaggg cuaAAAUCCCCUUCUUA UCA A**

**GUUUUA GCCUCCCUUAACCUCCC GAUUUUAGGGGAAGAAU AGU A**

**U^ -- A A U**

**zma-miRn8d GGAGGGAAUUGGAGGGGCUA**

**CC -| a g AA A**

**UCAAAAA Uggaggg auuggaggg cuaaAAU CCUCCUUAUUCA A**

**GGUUUUU GUCUCCC UAACCUCCC GAUUUUA GGAGGAAUAAGU A**

**CA G^ C G GG U**

**zma-miRn8e GGAGGGAAUUGGAGGGGCUA**

**CG-| C ga AUCUUCUCAUCCAA**

**ACAAAAU ggagg auuggaggggcuaGAAUC \**

**UGUUUUG CCUUC UAACCUCCCCGAUCUUAG A**

**CGG^ U UC AAAAAAAAUAAAUA**

**zma-miRn8f GGAGGGAAUUGGAGGGGCUA**

**UC| C c - C A**

**CAAAACC ggagggaauuggagggg uaAAAUCC CCUC UUAUUCA U**

**GUUUUGG UCUCCCUUAACUUCCCC AUUUUAGG GGAG AAUAAGU U**

**GU^ - U U - U**

**zma-miRn9a AUGCACUAGAGCUAAUGGUUA**

**- - --| A**

**GGGUGUUUGA augcacuagagcuaaugguua G UGGCUAAAA UUGC C**

**CCCACAAACUUACGUGAUCUCGAUUAUCAAUC AUCGAUUUU AACG U**

**C A UA^ A**

**zma-miRn9b AUGCACUAGAGCUAAUGGUUA**

**.-G ---- -| g U GUUA AAA**

**GGU GUUUGAau gcacuaga cuauugguuaGC AGCUAAUAA CUAAC \**

**UCG UAGAUUUA CGUGGUUU GAUUAUCAAUUG UCGAUUAUU GGUUG A**

**\ - AUGU U^ - - AAUC AUA**

**zma-miRn9c F1-m0153-3p AUGCACUAGAGCUAAUGGUUA**

**F1-m0153-5p ACUAUUAGCUCUAGUGCAUUC**

**A C .-CCCCCUAACUAUUA| U UGC**

**UUUUAG AGCUAaauauuagcucuagugcauucAAACA GC CUAG \**

**AAAAUC UCGauugguaaucgagaucacguaAGUUUGU CG GAUC A**

**- A \ --------------^ U UCG**

**zma-miRn9d** **AUGCACUAGAGCUAAUGGUUA**

**U A - -| CCUAACUA**

**GUUAGCUA CUAACUAUUAGCUCUA UGCAUUCAAA CAC \**

**UAAUCGAU Gauugguaaucgagau acguaAGUUU GUG U**

**- C c C^ AUCUUUAU**

**zma-miRn10a AACUAAUUGUGGGUCGUGUCGU**

**- ----| a GGCCAAUGAC C**

**UCACA CAUAUa cuaauuguggucggugucguG UUGGCA A**

**GGUGU GUGUAU GAUUAAUACCCAGCACAGCAC GAUCGU C**

**G UUUC^ A ---------- G**

**zma-miRn10b** **AACUAAUUGUGGGUCGUGUCGU**

**- - .-AUaa| GG GAUGACUG C**

**GGCAC ACC cuaauugugggucgugucgu GCC GGCAC \**

**CCGUG UGG GAUUAAUACCCAGCACGGCA CGG CCGUG A**

**U C \ ----^ -- -------- C**

**zma-miRn11 AUGGAGAGAACUGAGAAGGUAA**

**-| C aac aa a CC A AA**

**AG AAGUGUAauggagag ugag ggu aGAAU CUUA UAUUC \**

**UC UUCACAUUACCUCUC ACUC CCA UCUUA GAAU AUAAG A**

**C^ A CUA C- A UA A UU**

**zma-miRn12 ACUGGCUCACCAUCGUCUGGC**

**U| A AGC G G G U**

**UGUUAAGGGGCCAGACGAUGGUGAGCCGGUGUACU UGUCCGGAUGUGUGGUCACAAAAUUUAAAGCUGCCCU UUCAU UGUGCGCUUCUCUUCUCUUA GU GGCUGCUAAG G**

**ACAAUUCCCcggucugcuaccacucggucaCAUGA ACAGGCCUACACACCAGUGUUUUAAAUUUCGGCGGGA AAGUA ACACGUGAAGAGAAGAGAAU CA CCGACGAUUC A**

**A^ G CC- G A G U**

**zma-miRn13a UUGGAGGGGAUUGAGGGGGCUA**

**- -| U A g AAUAGGA**

**GA CAA GG Auuggaggggauugaggg gcuaG \**

**CU GUU CC UAACUUCCCCUAACUCCC CGAUC A**

**C C^ C C A CUAGGGG**

**zma-miRn13b UUGGAGGGGAUUGAGGGGGCUA**

**-| gg AU G AAUU**

**AGCACGGGGAuuggaggggauuga gggcuaAA AUCCCCUUU CUA \**

**UCGUGCCCCUAACCUCCCCUAACU CCCGAUUU UAGGGGAAA GAU U**

**C^ AA -- - AAGU**

**zma-miRn13c UUGGAGGGGAUUGAGGGGGCUA**

**-| AU G AAUU**

**AGCACGGGGAuuggaggggauugagggggcuaAA AUCCCCUUU CUA \**

**UCGUGCCCCUAACCUCCCCUAACUCCCCCGAUUU UAGGGGAAA GAU U**

**C^ -- - AAGU**

**zma-miRn13d UUGGAGGGGAUUGAGGGGGCUA**

**- g a -| AA**

**AGCAAGGGGAuug agggg uuga gggggcuaGAGU \**

**UCGUUUCCCUAAC UCCCC AACU CCCCCGAUCUUA G**

**C A C C^ GG**

**zma-miRn14a F1-m0104-5p UAACUAGCCGUCGGAGAUAAG**

**F1-m0104-3p UUAUCUCCGACGGCUAGUUAC**

**CA ACCG UU AAA -| A**

**GG UG AA AG uuuaucuccgacggcuaguuacGAGGCUGUCGGAUAUA C**

**CC AC UU UC gaauagaggcugccgaucaauGCUCCGGCAGCCUGUAU C**

**AC GAGA CU AUA U^ U**

**zma-miRn14b F1-m0288-3p UAACUAGCCGUCGGAGAUAAG**

**F1-m0288-5p UUAUCUCCGACGGCUAGUUAC**

**C ACCG UU AAA -| A A**

**GGG UG AA AG CuuaucuccgacggcuaguuacGAG CCGUCGGAUAUA C**

**CCC GC UU UC gaauagaggcugccgaucaauGCUC GGCAGCCUGUAU C**

**A GAGA CU AUA U^ C U**

**zma-miRn14c F1-m0666-3p UAACUAGCCGUCGGAGAUAAG**

**F1-m0666-5p UUAUCUCCGACGGCUAGUUAC**

**1**

**C ACCG UU AAA -| A**

**GGG UG AA AG cuuaucuccgacggcuaguuacGAGGCCGUCGGAUAUA C**

**CCC GC UU UC gaauagaggcugccgaucaauGCUCUGGCAGCCUGUAU C**

**A GAGA CU AUA U^ U**

**zma-miRn15a AAUCCAUAUGGAUUGGGGUUGA**

**U A AU- U G - .-UAUGGAUU| AGAU**

**UUGUUCG UU UUCCA UCCAUAUGGAUU GAGGG GAUUGA GUGAG \**

**AACAAGC Aa ggggu agguauaccuaa CUCCU CUAACU UAUUC U**

**G A guu u - A \ --------^ AGUU**

**zma-miRn15b** **AAUCCAUAUGGAUUGGGGUUGA**

**UGG------- G A A UG - A**

**GAUU AUAUGGAUUGGG GAG UUU A CUU U**

**uuag uauaccuaaCUC CUC AAA U GGG U**

**AAaguugggg G C C GU A A**

**zma-miRn16a UAUAUAAGUUGGAUUAUGGUA**

**- C-- aAAC A A -| AC C ACA**

**UCUC UAGAUuauauaaguuggauuauggu GG UAGGAGGGUA AAUAUCACUUUG GA CACAAAUAGGUUGAAA AAGCUA U**

**AGGG AUCUAAUAUAUUCAAUCUAAUACUA CC AUCCUUCUAU UUAUGGUGAAAC CU GUGUUUAUUCGACUUU UUCGAU U**

**A AAU CCUU C C A^ A- A GGU**

**zma-miRn16b UAUAUAAGUUGGAUUAUGGUA**

**G| U a g aAAAACG GA CAC ACCAC**

**CUCCUA AUuauaua guug auuauggu UAGGA GUAAAAUAU UUUAGU \**

**GAGGAU UAAUAUAU CAAC UAAUACCA AUCCU CAUUUUGUA AAGUCG A**

**A^ C A A CCUUCCA AC --- AAUAA**

**zma-miRn16c UAUAUAAGUUGGAUUAUGGUA**

**C -- aAAAAAAAUAG C----| C CUC AG CC UC A CCC**

**CUCCUAGAU uauauaaguuggauuauggu GAGGGUA AAUA UA UG AUCA AAUAA UGAAAUA GCUA A**

**GAGGAUCUA AUAUAUUCAACCUAAUACCA CUCCCAU UUAU AU AC UGGU UUAUU ACUUUAU CGAU A**

**A AC ACUUCACAUA- UUUUU^ A AAA CU AU CA C CGU**

**zma-miRn16d** **UAUAUAAGUUGGAUUAUGGUA**

**- G AG - A C- .-AU| CUC**

**CCUCCUA AUUAUAUAAGUUGGAUUAUGGU AAGGAUA GAAG GUAAA AUCACUUUGGGACC AAAUAAGCUA A**

**GGAGGAU UAAUAUAUUCGAUCUAAUACCG UUCCUAU CUUC CAUUU UAGUGAAACCUUGG UUUAUUUGAU A**

**A A CA C - UA \ --^ CGU**

**zma-miRn17 AAAAUUAGUCGAGAGGUUUA**

**A| G U AAA AAC U**

**GA UCU GUUCUAAAUCUCU ACUAAU UAG \**

**CU AGG UAAGauuuggaga ugauua aUC U**

**A^ - - gc- aa- G**

**zma-miRn18a UUUGGAUGCACUAGAGCUAAU**

**A .-GG c g U G ACU U AA- .-AACAAA| CUA**

**CAGGG UGuuuggaugca ua agcuaauAGUUA GU GCUAAAA GCUAG GG UUAGAUGGUU UAGCUAG \**

**GUCUU ACAAACUUACGU GU UCGAUUAUCAAU CG CGAUUUU CGAUU CU GGUUUGCCAG AUCGAUU A**

**A \ -- A A - A AAU U GUA \ ------^ AUC**

**zma-miRn18b UUUGGAUGCACUAGAGCUAA**

**AA G CU- .-UUA| CAUCCAAACAGUC UU**

**GGGGGGUGuuuggaugcacuagagcuaaUA UUAG GCUAAAA GCUGAAGG UAGCUAAUAG \**

**UCCCCCACAAACUUACGUGAUCUCGAUUAU AAUC CGAUUUU CGAUUUUC AUCGAUUAUC C**

**AA A AAC \ ---^ ------------- AA**

**zma-miRn19 GAAAUGUUGUCUGGUUCAAGG**

**- --- Uga uu a UUUGCG --------| UC C**

**GCU UUUGAG aaug gucugguuc aggUCUUGC AUUUGAGGA UGA CAUG U**

**CGA GGACUC UUAC CGGACCAGG UCUAGAACG UAGGCUCCU GUU GUAC U**

**U UAA CCC UU C UA---- UAUUUUUU^ GU G**

**zma-miRn20a UUUGUUAAUGUUUGGAGUAGC**

**-- GGA- C ACACACC CC - U- AA--| A**

**UCU UAAUuuuguuaauguuuggaguagg CCAUCAAAG AGUUC GU GU UCC AGCC C**

**GGA AUUAAAACAAUUACAAACCUCAUCG GGUAGUUUC UUAAG CG CA AGG UCGG C**

**CG AACA C CAAAU-- A- U UU ACCA^ A**

**zma-miRn20b UUUGUUAAUGUUUGGAGUAGC**

**- GGA- C A .-AC| CAG**

**UCU UAAUuuuguuaauguuuggaguagc CC UCAAAG ACAC U**

**GGA AUUAAAACAAUUACAAACCUCAUCG GG AGUUUC UGUG U**

**G AAUA C C \ --^ CCC**

**zma-miRn20c UUUGUUAAUGUUUGGAGUAGC**

**CUUUAU| G UUUAAAAUC G - .-GU U**

**UAAUuuuguuaauguuuggaguagc CCGUCAAAGG UGCA UAGU CCUG AGCC G**

**AUUAAAACAAUUACAAACCUCAUCG GGUAGUUUCU GUGU GUCA GGGC UCGG G**

**AGACCU^ G --------- G A \ -- U**

**zma-miRn20d UUUGUUAAUGUUUGGAGUAGC**

**-- GGA- C A ACACACC CCGU U- AA--| A**

**UCU UAAUuuuguuaauguuuggaguagc CC UCAAAG AGUUC GU UCC AGCC C**

**GGA AUUAAAACAAUUACAAACCUCAUCG GG AGUUUC UUAAG CA AGG UCGG C**

**CG AACA C C CAAAU-- AUGU UC ACCA^ A**

**zma-miRn20e UUUGUUAAUGUUUGGAGUAGC**

**C U-| G UUUAAAUUC G - .-GU U**

**UUUG UAAUuuuguuaauguuuggaguagc CCGUCAAAGG UACA UAGU CCUG AGCC G**

**AGAC AUUAAAACAAUUACAAACCUCAUCG GGUAGUUUCU GUGU GUCA GGGC UCGG G**

**A CU^ G --------- G A \ -- U**

**zma-miRn20f UUUGUUAAUGUUUGGAGUAGC**

**C U-| G UUUAAAUUU G - .-GU U**

**UUUG UAAUuuuguuaauguuuggaguagc CCGUCAAAGG UGCA UAGU CCUG AGCC G**

**AGAC AUUAAAACAAUUACAAACCUCAUCG GGUAGUUUCU GUGU GUCA GGGC UCGG G**

**A CU^ G --------- G A \ -- U**

**zma-miRn20g UUUGUUAAUGUUUGGAGUAGC**

**-- GGA- C A .-AC| CAG**

**UCU UAAUuuuguuaauguuuggaguagc CC UCAAAG ACAC U**

**GGA AUUAAAACAAUUACAAACCUCAUCG GG AGUUUC UGUG U**

**CG AACA C C \ --^ CCC**

**zma-miRn21a UUGAGCCGCGUCAAUAUCUCC**

**G U C--| C AG A G**

**ACAA GC GGGAGGUAUUGGCGUG CUCAAUCCGA GCGUGGCUG CUG \**

**UGUU CG Cccucuauaacugcgc gaguuAGGUU UGUACCGAC GGC C**

**- - UUC^ c CU - G**

**zma-miRn20b UUGAGCCGCGUCAAUAUCUCC**

**C| A G CG C AG AUAGAU**

**UAGG CAA GG GAGGUAUUGGCGCG CUCAAUCCGA GCGUGGCUG U**

**GUCC GUU Cc cucuauaacugcgc gaguuAGGUU UGUACCGAC G**

**U^ - G -- c CU GGCGCG**

**zma-miRn22a AAAAGAAACGGAUGGAGUAUA**

**AUAAAC------ AUC G A**

**UACUCCAUCCGUUUC UUAGUUGUCGCUGGAUAGU CA A**

**augagguaggcaaag aAUUAACAGCGACCUAUCA GU A**

**GAUAUACCGUau aaa A U**

**zma-miRn22b AAAAGAAACGGAUGGAGUAUA**

**AAGCAAAAUA| C G G A**

**UAUACUCC UCCGUUUC UUUUAGUUGUCGCUGGAUAGU CA A**

**auaugagg aggcaaag aaaaUUAACAGCGACCUAUCA GU A**

**UGACAAAUGA^ u a A U**

**zma-miRn22c AAAAGAAACGGAUGGAGUAUA**

**ACAA G A C G .-AAC| UCG U**

**CUAUU UA ACUCC UCCGUUUC UUUUA GUCG CUA U**

**GAUAG au ugagg aggcaaag aaaaU CGGU GAU U**

**UCAG A a u a \ ---^ UA- A**

**zma-miRn22d AAAAGAAACGGAUGGAGUAUA**

**CAGCUAA| A C G A GCAA**

**UCA AUACUCC UUCGUUUC UUUUAGUUGUCGCUGG UAGU A**

**AGU uaugagg aggcaaag aaaAUUAACAGCGACC AUCA A**

**UGCCAAC^ a u a C AAUU**

**zma-miRn23a UGGAGGGGAUUGAGGGGCAUA**

**-| A a A C U U**

**GCAUGGGGA uggaggggauugaggggc uaUAAUCCC UG UG UCA U**

**CGUACCCUU ACCUCUCCUAACUCCCCG AUAUUAGGG AC AU AGU U**

**A^ A G A A C U**

**zma-miRn23b UGGAGGGGAUUGAGGGGCAUA**

**AAU C -| C UU CUC - CC U C**

**GGGAUU UA GCCCCUC AAU CUUCCGAUUUU GC AC AAAU AGUC U**

**CUCUAA au cggggag uua ggagguUAAGG CG UG UUUA UCGG A**

**UCU U a^ - gg AUA C A- U A**

**zma-miRn23c UGGAGGGGAUUGAGGGGCAUA**

**AACAA--| GA A GUC**

**GA UuggaggggauugaggggcauaUAAUUC UUGCUA A**

**CU AAUCUCCUCUAAUUCCCCGUAUAUUAAG AACGAU U**

**AGGUACC^ G- G AAG**

**zma-miRn24 UCACAAUCGAUUGGACUAAAA**

**U GA GC U C .-AUGU| U U UC**

**UCU UGUCAUUUUAGUCCAAUCG UGUGAACA GAC UGG GGCU UGCCA CGUG \**

**AGG ACAGUaaaaucagguuagc acacuUGU CUG ACC CCGG ACGGU GCAC G**

**- UC ua C A \ ----^ U - CU**

**zma-miRn25a AAUAAAAAGAAACGGAUGGAG**

**CAAAUAU| U CAA**

**GUACUUC UCCGUUUCUUUUUAUUUGUCGCUGGAUAGUG U**

**UAUgagg aggcaaagaaaaauaaACAGCGACCUAUCAC U**

**GACGACC^ u AUU**

**zma-miRn25b AAUAAAAAGAAACGGAUGGAG**

**AAAAAAAU| C A**

**UACUCC UCCGUUUCUUUUUAUUUGUCGCUGGAUAGUGUA A**

**AUgagg aggcaaagaaaaauaaACAGCGACCUAUCACGU A**

**UUUUAUUC^ u U**

**zma-miRn25c AAUAAAAAGAAACGGAUGGAG**

**U| GUCAA C C AA A CGU ACAA**

**UUG UACUCC UCCGUU CUU UAU UGUCGUC UAGU A**

**AAC AUgagg aggcaa gaa aua ACAGCAG AUCA U**

**-^ AAUAC u a aa a CUC AAUU**

**zma-miRn25d AAUAAAAAGAAACGGAUGGAG**

**G| CUCCAAACUGA C CAA**

**CUGCAUG UACUCC UCCGUUUCUUUUUAUUUGUCGCUGGAUAGUG A**

**GACGUAC AUgagg aggcaaagaaaaauaaACAGCGACCUAUCAC A**

**A^ ----------- u AUU**

**zma-miRn25e** **AAUAAAAAGAAACGGAUGGAG**

**UGGUA -| C A**

**UG UACUCC UCCGUUUCUUUUUAUUUGUCGCUGGAUAGUGUA A**

**AC AUgagg aggcaaagaaaaauaaACAGCGACCUAUCACGU A**

**AUAAA G^ u U**

**zma-miRn25f** **AAUAAAAAGAAACGGAUGGAG**

**UAUAUAUA| C UAA**

**UACUCC UCCGUUUCUUUUUAUUUGUCGCUGGAUAGUG A**

**AUgagg aggcaaagaaaaauaaACAGCGACCUAUCAU A**

**AUCCUACC^ u GUU**

**zma-miRn26a GGGGAUUGAAGAGGAUUAAAU**

**- ----------| a A A**

**UCGGUUGCUA ggggauugaag ggauuaaauCCCC CCUAUUCA U**

**AGCCAAUGAU CCCCUAACUUC CCUAAUUUAGGGG GGAUAAGU U**

**A CACCAAACUC^ C A U**

**zma-miRn26b GGGGAUUGAAGAGGAUUAAA**

**-| a CG**

**CAAUCCAGAgggggauugaag ggauuaaA G**

**GUUAGGUCUCCCCCUAACUUC CCUAAUUU G**

**G^ C AG**

**zma-miRn26c GGGGAUUGAAGAGGAUUAAAU**

**- -| A GA g AA**

**GG GG UUG ggggauugaaga gauuaaauCCCCUCCUAUUC \**

**CC CC AAC UCCCUAACUUCU CUAAUUUAGGGGAGGAUAAG A**

**A A^ A UC A UA**

**zma-miRn26d GGGGAUUGAAGAGGAUUAAAU**

**GGGGAUUGGA| a U A**

**ggggauugaag ggauuaaauCCCCUCCUAU CA U**

**CCCCUAACUUC CCUAAUUUAGGGGAGGAUA GU U**

**C---------^ C C U**

**zma-miRn26e GGGGAUUGAAGAGGAUUAAAU**

**CUA -----------| aga UCA GUCAA**

**GGUUGU Aggggauuga ggauuaaauCCC CUA A**

**CCAACG UUCCCUAGCU CCUAAUUUAGGG GAU A**

**AAA CAGACCUAGCU^ CCC UAA AAAUU**

**zma-miRn26f GGGGAUUGAAGAGGAUUAAAU**

**- .-AUUG -| A**

**GAGG GAgg ggauugaagaggauuaaauCCCCUCCUAUUCA U**

**CUCC CUCC CCUAACUUCUCCUAAUUUAGGGGAGGAUAAGU U**

**A \ ---- U^ U**

**zma-miRn26g GGGGAUUGAAGAGGAUUAAAU**

**C| GCA g - ga C U A**

**GGAU AG gggauugaa gag uuaaauCCC UUCUA UCA A**

**CCUA UC CUCUAACUU CUC AAUUUAGGG AAGAU AGU U**

**A^ AC- A U A- A C U**

**zma-miRn26h** **GGGGAUUGAAGAGGAUUAAAU**

**GGGGAUUGGA| a C A**

**ggggauugaag ggauuaaauCCC UCCUAUUCA U**

**CCCCUAACUUC CCUAAUUUAGGG AGGAUAAGU U**

**C---------^ C A U**

**zma-miRn26i** **GGGGAUUGAAGAGGAUUAAAU**

**UC| g a aga AU A**

**GGUUGCUAg gg uuga ggauuaaauC CUUCUAUUCA U**

**CCAAUGAUC CC AACU CCUAAUUUAG GAGGAUAAGU U**

**AA^ A A CCC GG U**

**zma-miRn26j** **GGGGAUUGAAGAGGAUUAAAU**

**- ----------| a CC A**

**UCGGUUGCU Aggggauugaag ggauuaaauC CUCCUAUUCA U**

**AGCCAAUGA UCCCCUAACUUC CCUAAUUUAG GAGGAUAAGU U**

**A UCACCAAACU^ C AA U**

**zma-miRn26k** **GGGGAUUGAAGAGGAUUAAAU**

**- -| A GA a A**

**GG GG UUG ggggauugaag ggauuaaauUCCCUCCUAUUCA A**

**UC CC AAC UCCCUAACUUC CCUAAUUUAGGGGAGGAUAAGU A**

**A A^ A UC C U**

**zma-miRn27 UUUGGGGUGGAUACGUGGUCA**

**CC C U | GCAG**

**UGUUUGGAUG CCAUGUAUCCACCUCAA CCAUGU--GUGGAAU \**

**ACAAACCUac ggugcauaggugggguu GGUACA CACCUUA U**

**GA u u \ ^ AAUU**

**zma-miRn28 UAGUCGACGAUCAAUGGCCAC**

**- c A AAAAUA .-CAAUAAUAUC| ACA**

**UACGAGCCUCuagucga gaucaauggccac CAACCCCCAUUUU--UGUCA ACAUUUU AAAGUUCA \**

**AUGCUCGGAGGUCAGCU CUAGUUACCGGUG GUUGGGGGUAAGA ACGGU UGUAAGA UUUUAAGU C**

**C A C \ AUCUAG \ ----------^ AUC**

**zma-miRn29a AAUUCAUAUGGAUUGGGGUA**

**U C AU-|UU A UACGGAUUG GA UG - A**

**UGUU GGUU U CAAUCCAUAUGGAUUGA GGGGAUUGA GAGGG UUU A CUU C**

**ACAA CCAA g guuagguauacuuaaCU CCCCUAACU CUCCC AAA U GAG U**

**- A Gau^gg C --------- -- GU A A**

**zma-miRn29b AAUUCAUAUGGAUUGGGGUA**

**U AU- UU GA .-UACGGAU| AAAAA**

**UGUUCGGUU U CAAUCCAUAUGGAUUG GGGGGUUGA UGAG \**

**ACAAGCCAA g guuagguauacuuaAC CCCCUAACU AUUC U**

**- Gau gg UC \ -------^ AGUUG**

**zma-miRn29c AAUUCAUAUGGAUUGGGGUA**

**U AU-|UU GA UACGGAUU A A UAA A**

**UGUUCGGUU U CAAUCCAUAUGGAUUG GGGGAUUGA GG GG GAUUU CUU C**

**ACAAGCCAA g guuagguauacuuaaC CCCCUAACU CC CC CUAAG GGG U**

**- Gau^gg UC -------- - - UUA A**

**zma-miRn29d AAUUCAUAUGGAUUGAGGUA**

**U AU-| U - UAU AUU GA- GACU**

**UGUUCGGUU UUUCAAUCCAUAUGGAUU GAG GGGAUUGA GG GGGA GAUUUU \**

**ACAAGCUAA ggaguuagguauacuuaa CUC CCCUAAUU UC CCCU UUAGGA U**

**- Gau^ - U --- --- AAA AUGA**

**zma-miRn30 AGUUCUUGUUGGCCAUGUUC**

**C ----| C G**

**AAGACAUCCAGAACAUGGCCAACAAGAACUGGUGCAAAGCGGUAGUUGACAGGC AGAACAUGGCCAA AAGAACUGGUGCAAAGCGGUAGUUGAC C**

**UUCUGUAGGUcuuguaccgguuguucuugaCCACGUUUCGCCAUCAACUGUCCG UCUUGUACCGGUU UUCUUGACCACGUUUCGCCAUCAACUG C**

**- UAGG^ A U**

**zma-miRn31a F1-m0239-3p UUGGAUUUUGAUUGGAUGCAC**

**F1-m0239-5p GCAUCCAAUCAAAAUCUAACA**

**CCG- .-AA C - G GG G**

**GUgcauccaaucaaaaucuaac UUA UG CUAUUUUA GU AA A**

**cacguagguuaguuuuagguuG AAU AC GAUAAAAU UA UU G**

**GUCA \ -- U A G AA G**

**zma-miRn31b UUGGAUUUUGAUUGGAUGCAC**

**C G U .-GAUGGUU| GG U**

**AAGU GUC GGUGCAUUCAAUCAAAAUCCAAC GUUAUUUUACGU AA G**

**UUCA UAG UcacguagguuaguuuuagguuG CAAUAAAAUGUA UU G**

**- A C \ -------^ AA G**

**zma-miRn31c UUGGAUUUUGAUUGGAUGCAC**

**GU AAGCUAUA CA | GG**

**GCAAUC UGGUGCAUCUAAUUAAAAUCCAA GUGACUGCUAU--UUUAGCU \**

**CGUUAG ACcacguagguuaguuuuagguu CACUGACGAUA AAAUUGG A**

**UU -------- AC \ ^ GA**

**zma-miRn31d**  **UUGGAUUUUGAUUGGAUGCAC**

**A - G C ACA GG--| AA**

**AGUG AU UGGUGCAUCUAAUCAAAAUUCAA GGUGGC UAUUUUA UAG G**

**UUAC UA ACcacguagguuaguuuuagguu CCAUUG AUAAAAU AUU G**

**- U G A ACG AUAA^ GG**

**zma-miRn32 ACGGAGUGGUAGUGGAGGGUAUG**

**C .-AUAGa ga - -- AG UG UC .-AUGU UACUAAC G C .-GC| GA**

**AUGUC cg gugguaguggagggua ugC GCG GG AGG GA UCGAU AACCGC UAG GC--GUGAAAAAU CGC \**

**UACAG GC CACCGUCAUUUUCUAU ACG CGC CU UCC CU AGCUG UUGGCG GUC CG CAUUUUUUA GCG C**

**U \ ----- AC G UA CA GA UA \ ---- UAUUAUU - A \ \ --^ UU**

**zma-miRn33 UCGGACCAGGCUUCAUUCCCCG**

**U| UCU A C C ACG A- U UG**

**CAUG GUCG GGGGAAUGA GUC GGUCCGA AGCC CGGC GC C**

**GUAC CAGU ccccuuacu cgg ccaggcu UCGG GCCG CG U**

**G^ U-- g u a --- GC C CG**

**zma-miRn34a UUUGAAUGCACUAGAGCUGAU**

**UAA - - G A- ----- | G**

**GAGG G UGuuugaaugcacuagagcugauA UUAGU GCUAAA AUUAGU--UGGA A**

**UUCC C ACAAACUUACGUGAUCUCGAUUAU AAUCG CGAUUU UGAUCA ACCU C**

**UGA A A A AC UUUAA \ ^ A**

**zma-miRn34b UUUGAAUGCACUAGAGCUGAU**

**UAAGC a c G .-GGCUAAAAAAUU .-AAAUUA| AC**

**GGGUGuuugaaugcacu gag ugauA UUAGUU GCUAGUA GCUAGCUA \**

**CCCACAAACUUACGUGA CUC AUUAU AAUCGA CGAUUAU CGAUCGAU A**

**GAAUU C C G \ ------------ \ ------^ AA**

**zma-miRn34c UUUGAAUGCACUAGAGCUGAU**

**A AC-- AUA -| UA**

**GCUC UAUUAGCUCUA CAUUCAAACACUUC CU A**

**CGGG Auagucgagau guaaguuuGUGAGG GA U**

**A GAUC cac U^ UU**

**zma-miRn35 AUGCAGAACAAUUUACAGACG**

**-| C a g cg A AUUUCUCA GCA**

**UAG AUUCUU ugca aacaauuuacaga GAAAUG UUUGUACU GGU A**

**AUC UAAGAA ACGU UUGUUGAAUGUCU CUUUAC AAACGUGG CCA A**

**C^ A C G AG C ACUCUUUA ACC**

**zma-miRn36a UGUGGAUUAGGUGGGAUUGGA**

**40**

**- A a U-----------| C U CA**

**UCUUA UUCAuguggauu ggugggauugga GGAUUU AA CU A**

**AGGAU GGGUACACCUAA CUACCCUAACCU UCUAAA UU GA A**

**A A C UUUUUAAUACUU^ A - AC**

**zma-miRn36b UGUGGAUUAGGUGGGAUUGGA**

**- CAA a | UUU**

**UCU UCCAuguggauu ggugggauugga--CGGG \**

**AGG AGGUACACCUAA CUAUCCUAACUU GCCC A**

**A AC- C \ ^ UAA**

**zma-miRn36c UGUGGAUUAGGUGGGAUUGGA**

**- CAA a | UUU**

**UCU UCCAuguggauu ggugggauugga--UGGG \**

**AGG GGGUACACCUAA CUACCCUAACCU ACCC C**

**A AUA C \ ^ UAA**

**zma-miRn36d** **UGUGGAUUAGGUGGGAUUGGA**

**- CAA a U-| UCAAUCCUAAAUAA**

**UCU UCCAuguggauu ggugggauugga GAGUU \**

**AGG GGGUGCACCUAA CUACCCUAACCU UUUAA G**

**A AUA C UU^ UACUUUCUAAAACU**

**zma-miRn37a AGACUUAGGAACGGAGGGAGU**

**GAAAAUAA U .-AAGCAAAUU .-AAAAAUCU| GU**

**GUACUCCCUCCGUUCCUAAGUCUCCA CGUAU GGA--UUUAUUC CCAUC A**

**CAugagggaggcaaggauucagaGGU GCAUA CCU AAAUGAG GGUGG U**

**GAACACUA C \ --------- \ \ --------^ AU**

**zma-miRn37b AGACUUAGGAACGGAGGGAGU**

**UCUU U G U .-C G CA ACCA .-A| CAA CC**

**GUGA GUACUCCCUCCGUUCCUAAGUCUCCA CGUAU AGU AUCU CA GUG AGG GCGG AGGAGCG G**

**UAUU CAugagggaggcaaggauucagaGGU GCAUA UCG UAGA GU CAU UCC CGCU UUUUCGU A**

**CUUU - A U \ - G AG AA-- \ -^ CA- AU**

**zma-miRn38 UUUGAAUGCACUAGAACUAAUU**

**UU A a ac u UA AAAU .-AG| C AACAAAU**

**AG GGGUGuuugaaugc cuaga uaau GUUAGU GCUAAA GUUAGU AAUUAG UAGCC \**

**UC CCCACAAACUUACG GAUCU AUUA CAAUCA CGAUUU UAAUCA UUAAUU AUCGG A**

**AU C C CU U C- ---- \ --^ - CCAAUCA**

**zma-miRn39 UUUGGCAAAAUGGGUCGCUCUA**

**GG A UU UU**

**AGGGCGACCCAUUUUG CAAAU CUC \**

**ucucgcuggguaaaac guuuA GAG C**

**Aa g GG CC**

**zma-miRn40a AAGGAACCGCCAGUGGAAAUGCU**

**- GC -| a c au uC UU CUCU**

**CGGUUG UUaag ga ccgc aguggaa gc UUCCACUG GUGGCU U**

**GCCGAC AAUUC CU GGUG UCACCUU CG AAGGUGAC CGCCGA A**

**C UC U^ C A CU UA -- CUCA**

**zma-miRn40b AAGGAACCGCCAGUGGAAAUGCU**

**- GC -| a c au uC UU CUCU**

**CGGUUG UUaag ga ccgc aguggaa gc UUCCACUG GUGGCU U**

**GCCGAC AAUUC CU GGUG UCACCUU CG AAGGUGAC CGCCGA A**

**C UC U^ C A CU UA -- CUCA**

**zma-miRn40c AAGGAACCGCCAGUGGAAAUGCU**

**C U -- ---| a g AU U CUCUCU**

**GG UGG CUUa agga ccgccagug aaaugcu UUUCACU GCGG U**

**CC ACC GAGU UCCU GGCGGUCAU UUUACGA AAAGUGA CGCC A**

**A - AA AUA^ - G -- C AACACA**

**zma-miRn40d AAGGAACCGCCAGUGGAAAUGCU**

**C c AU----| UC CGG**

**GGUUGGCUUAaggaaccgccaguggaaaug uAUUUUCACUAGC CUU CAC U**

**UCGGCCGAAUUCCUUGGCGGUCACCUUUGU AUAAAGGUGAUCG GAA GUG G**

**C - CCAAGU^ UU UUG**

**zma-miRn41 ACAUUUUAAAUCUGGGACAUC**

**- c CAG AGC CACC-| G CC CCU**

**GCUCACGUGCacauuuuaaaucugggacau CAUC CAUCUAAC AG AAUU UAUAAAGAACUCCUUCCA UC C**

**CGAGUGCACGUGUAAAAUUUAGACUCUGUA GUAG GUAGGUUG UC UUAA AUGUUUUUUGAGGAGGGU AG C**

**A A AAA CCA CAAGA^ A -- UAC**

**zma-miRn42 UGAAGACUAAACGACGAGACA**

**AUA| a cg aAA C UUAAA CG AAGAA UACUU**

**ACAUAGAuga gacuaaacga agac UUUAUUAAG CUAA UCCA AUU AUGUG \**

**UGUGUCUAUU CUGAUUUGCU UCUG AGAUAAUUC GAUU GGGU UAA UACAC U**

**UAA^ C AU CUC A C---- AA GCGAG CACGA**

**zma-miRn43 AGUGAGACCCUGCAGAAGCUGC**

**G --| U GAU U- A CU**

**CUGCU GGUGU GCU UCUGC GGUUUUACUG AUUUG \**

**GGCGA CUACG cga agacg ccagagugaU UAGAC A**

**- GU^ U --- uc G AC**

**zma-miRn44 GUCUUAAACACUCGGCAAAG**

**GCCGA UCC UUCA | UCCGA**

**GUGUUUU UUGCCGAGUGUUU ACACUCGGUAAAG--UCUUUGCCGAGUG \**

**CGCAAga aacggcucacaaa ugUGAGCCGUUUC AGAAACGGCUCAU A**

**CUUAG --- uuc- \ ^ GAAAA**

**zma-miRn45 F1-m0365-3p UGCCUUGGUCGCACGGUUGCA**

**F1-m0365-5p CAACCGUGCAACUAAGACACA**

**UU a a U .-AGAUG| AAAAA**

**CCUAUUGGUGcaaccgugc acuaag cacaaGGAU GAGGUG ACAUUUUU U**

**GGAUAACCacguuggcacg ugguuc guGUUUCUA UUUUAC UGUAGGGG A**

**AU c c U \ -----^ GAUUU**

**zma-miRn46a UUGGAUUGGUUUAGAGUGGUUC**

**UAUAAA| g u ag UU GCA**

**ACAGuu gauuggu uag ugguucAAC GC \**

**UGUCAA CUGACCA GUC GCCAAGUUG CG U**

**UUUAUG^ G C CG U- AUG**

**zma-miRn46b UUGGAUUGGUUUAGAGUGGUUC**

**UAUAAA| g u ag UU GCA**

**ACAGuu gauuggu uag ugguucAAC GC \**

**UGUCAA CUGACCA GUC GCCAAGUUG CG C**

**UUUAUG^ G C CG U- AUG**

**zma-miRn46c UUGGAUUGGUUUAGAGUGGUUC**

**UAUAAA| g u ag UU GCA**

**ACAGuu gauuggu uag ugguucAAC GC \**

**UGUCAA CUGACCA GUC GCCAAGUUG CG C**

**UUUAUG^ G C CG U- AUG**

**zma-miRn46d** **UUGGAUUGGUUUAGAGUGGUUC**

**UUUAAA g u ag --| G**

**ACAGuu gauuggu uag ugguucAAC UUGC C**

**UGUCAA CUGACCA GUC GCCAAGUUG GAUG A**

**UCUAUG G C CG UU^ C**

**zma-miRn47 GCUGGAAACGAGUCGAGCCGAG**

**CG .-A| gc aa U**

**CAAGU GG ugga cgagucgagccgagC \**

**GUUCG CC ACCU GCUCGGCUUGGCUCG C**

**CG \ -^ UU -- A**

**zma-miRn48a AUAUACUAUUAUGACCUCUAA**

**- A A A U UUCAU C C C UG | CAG G**

**CU UUUAC CauauacuauuaugaccucuaaACGA ACAGUA AUA UAAAGAAUGG UACUGAUAG AAC CG CGC--CCGCGCAUG GCACGCGU U**

**GA AAAUG GUAUAUGAUAAUACUGGAGAUUUGCU UGUCAU UAU AUUUUUUAUC AUGACUAUC UUG GU GCG GGUGUGUAC CGUGCGCG G**

**C G C G - ----- A A U GU \ ^ UA- U**

**zma-miRn48b AUAUACUAUUAUGACCUCUAA**

**- C C .-AUAUAAAAAAUA .-ACACACGCA| C**

**CU UUUACGCauauacuauuaugaccucuaaACGA ACAGUA GUUACUGAUAGUAAC GG C**

**GA AAAUGUGUAUAUGAUAAUACUGGAGAUUUGCU UGUCAU CGAUGACUAUCGUUG CC A**

**C U U \ ------------ \ ---------^ G**

**zma-miRn49a UUUCAAAGUUCGUGGACCUAA**

**UGA U C- U A C G C .-UAUCCCUAAACU| AA**

**GUGUCACUUA GUCCAC ACUUUGAAAAC CAUUUC GGGUC CUGAACU GUUAAGUGGUG ACUGCA UGUA A**

**UACAGUGaau caggug ugaaacuuuUG GUAAAG UCCAG GAUUUGA CAAUUCACCAC UGGCGU ACGU U**

**UCC c cu U A A A C \ ------------^ GA**

**zma-miRn49b UUUCAAAGUUCGUGGACCUAA**

**UCAA UGCCACUU - U C C A .-ACACUUCAC AAA-| U**

**GGGU GGUUCAUGAACUUUGAAAACA AUUUCU GUC UUAAA UUGUUAAGUG UGUACC UAGACUUGUA CAG G**

**UCCA ccaggugcuugaaacuuuUGU UAAAGA CAG GAUUU AACAAUUCAC ACGUGG AUCUGGGCGU GUC C**

**UCAA CUaau--- G U U A C \ --------- CGCC^ A**

**zma-miRn49c UUUCAAAGUUCGUGGACCUAA**

**AAGA C A ----- C .-A - GUAU G**

**AUG CACUUAGGUCCACGAACUUUGAAA UGUAUUUCUAGGUCCUUGAACUUGUUAAGUGA CACAC UC GUG GUCG GGUCCA A**

**UAC GUGaauccaggugcuugaaacuuu ACAUAAAGAUCCAGGAACUUGAACAAUUCACU GUGUG AG CAC UAGC CCAGGU A**

**CCC- A C AUAUG U \ - G AU-- A**

**zma-miRn50 UUCAAAGUUCAUGGACCUAAU**

**A| C C C U C U AU A CCCUAAACUU CACAU AU**

**AGGGAUG CA UUAGGUCUA GAACUUUGAAAAUGU UUUCUAGGCC UUGAACUUGU AAGUG GUACAU CAU GUAU GUCC \**

**UCCCUAC GU aauccaggu cuugaaacuuUUAUA AAAGAUCCGG AACUUGAACA UUCAC CGUGUA GUA CAUA CAGG U**

**-^ A u a U A U -- - UCACCAGC-- C---- UU**

**zma-miRn51 UUCUGGAGGGGAUUUGAGUUU**

**G| U U AG GC**

**GCUAGUUUGGAAACUCAAAUCCCUUCCGG AUUGGAGG GAUUGAGGGG AAU A**

**CGAUCAAACCuuugaguuuaggggagguc uAAUCUCC CUAACUCCUU UUA A**

**-^ u U CU AU**

**zma-miRn52 AUCGAAUGAGAUUGGGGGGAU**

**- C uga -| UC U A**

**UUGUG CUGGaucgaa gauugg ggggauUAAAUCU UUCUA UCA U**

**AAUGC GACCUAGCUU CUAACC CCUCUAAUUUAGA AGGAU AGU U**

**C A CCC U^ GA C U**

**zma-miRn53 CAAGCAAACUCAUCAACCUCAA**

**UG| CAA U U GG GCAAA U UGA**

**UGGAUGAC UGAGGUUG UGA GU GU GGUGG GGG C**

**ACCUACUG acuccaac acu ca cg UCACC UCU A**

**GA^ a-- u - aa aac-- - UGA**

**zma-miRn54 UAAGGCUUAUUUCCGUCGGUU**

**CGU| C G UA**

**CGGC AG AACCGACGGGAAUAAGCCUUAAACCGACGGGAAU \**

**GCCG UU uuggcugccuuuauucggaauUUGGUUGCCCUUA C**

**CU-^ A G UU**

**zma-miRn55 AAUUCAUAUGGAUUGAGGUA**

**U A AU-| AG AA UACAGAUU AGAGA - A**

**UGUUCG UU UUUCAAUCCAUAU AUUGG GGGAUUGA GGG UUUUGA CUU C**

**ACAAGU AA ggaguuagguaua uaaUU CCCUAACU CCC AAAAUU GAG U**

**- C Gau^ cu CC -------- ----- A A**

**zma-miRn56 UGAAGUGGAUUAGAGGGGCUA**

**G| A a u a c G CC A**

**CAAGGGAA ug ag ggauu gagggg uaAAAUC CCUU UAUUCA U**

**GUUCCCUU AC UC CUUAA CUCCCC AUUUUAG GGAA AUAAGU U**

**G^ - C C C A G CA U**

**zma-miRn57a UUUGACCAAGUUUGUAGAAAA**

**- AC ug CC - .-GAAAUUG| C AC**

**CUUCUCUA uuugaccaaguu uagaaaaAUAUAUUU AUCUAAGAUA CAAA CACUAU AAG \**

**GAAGAGAU AAACUGGUUUAA AUCUUUUUAUGUGGA UAGAUUUUGU GUUU GUGGUA UUC A**

**U AC GU UU A \ -------^ C AU**

**zma-miRn57b** **UUUGACCAAGUUUGUAGAAAA**

**CUUC C u aG C | AAAUAAAUUGCA A CA**

**UCUAA uuugaccaaguu guagaaa UACAUUAG AUCUA--UGAAAC CUAUCA GA U**

**AGAUU AAACUGGUUUAA UAUCUUU AUGUGGUU UGGAU ACUUUG GGUGGU CU A**

**GUAA C U CU U \ ^ GUACUGAUAUA- A UU**

**zma-miRn58a UGAGGGAGUGUUAGAGGGUCAG**

**UAG| U g g gg UUAUA C U**

**UAGCA Uugag gaguguua ag ucag CUU GUUGA \**

**AUCGU AAUUC UUCACAGU UC AGUC GAA CAAUU U**

**AGA^ U G - A- ----- A U**

**zma-miRn58b UGAGGGAGUGUUAGAGGGUCAG**

**CAA| U g a gg AUA C UU**

**UAGCA Uugag gaguguu gag ucag UA UUAG G**

**AUCGU AAUUC UUCACAG CUC AGUC AU AAUU A**

**AGA^ U G - A- GAG - UU**

**zma-miRn59a CCGGAGGGAUUGGAGGGGCUA**

**-| A AA c AUCCCAUUC**

**AGCC CAA ccggagggauuggagggg uaAA \**

**UCGG GUU GGCCUCCCUAAUCUCCCC AUUU U**

**C^ - CC - AAAACUUAU**

**zma-miRn59b CCGGAGGGAUUGGAGGGGCUA**

**- G A - g CC UGGC CA U U - .-CCAAAC| UCUU**

**AGCUA AAA ccgga gggauuggagg gcuaGAAUC CCUUCUUAUUUG CUC GA UUG GGC UC UAGC A**

**UCGGU UUU GGCCU CCCUAACUUCC CGAUCUUAG GGAAGAAUAAGU GAG CU AGC CCG AG GUCG U**

**C G A C A A- UAAA A- - - U \ ------^ UUGG**

**zma-miRn59c** **CCGGAGGGAUUGGAGGGGCUA**

**A .-AAAU au-| A**

**GUCC Accggaggg uggaggggcuaAAAUCCCCUCCUUAUUCA A**

**CAGG UGGCCUCCC ACUUCCCCGAUUUUAGGGGAGGAAUAAGU A**

**A \ ---- CUU^ U**

**zma-miRn60 CAAGAUCUAUGGCACCGACCC**

**G CA A G-| A CCG**

**CAAGUCCCAGGGGUCGG CCAUAGAUCUU GCGCCGACCCUCU GAC GCG A**

**GUUCAGGGUCcccagcc gguaucuagaa CGUGGCUGGGGGG CUG CGC A**

**- ac c AA^ C UCC**

**zma-miRn61a GCUUAUUUUCGGCGGCGUCUG**

**CGGAUUU CCA CA A**

**UUUUGGCGGCCAG CCGCCGAAAAUAAGCC UUUGCCGCCGAAAAUA \**

**AAAGCCGCCGguc ggcggcuuuuauucgg AAACGGCGGCUUUUAU C**

**------- ugc A- U**

**zma-miRn61b GCUUAUUUUCGGCGGCGUCUG**

**U| A A C A A**

**UUUUGGC GCCAGACGCCGCCGAAAAUAA CU UUUGCC CCGAAAAUA \**

**AAAACCG CGgucugcggcggcuuuuauu gG AAACGG GGCUUUUAU G**

**-^ C c U C C**

**zma-miRn62a UGGACAAGAUUUGAUGUUAGC**

**G A U A C C A AAC .-A| CGAUGUG UCUAU**

**GU CUAACAUGCUAACAUCAAAUC UUGUCUAUCGGAUC AU AUCUG GGUUGA UGU CCC UCUGAGGA CCCUAU \**

**CA GAUUGUAcgauuguaguuuag aacagguAGCCUAG UG UAGAU CCGAUU ACG GGG GGAUUUCU GGGAUG G**

**- C - G A A C CUA \-^ UA----- CAAAA**

**zma-miRn62b UGGACAAGAUUUGAUGUUAGC**

**G AC AA C --- .-GGGGAU| C G AUAU G**

**UG AACAUGCUAACAUCAAAUCUUGUCCAUC AUCCAUCAUCUA GGCUGAGUGCAAUC CCACCU AG AC CC UAUGU A**

**AC UUGUGcgauuguaguuuagaacagguAG UAGGUGGUAGAU CCGAUUCACGUUAG GGUGGA UC UG GG AUACA A**

**A GA CC A AGC \ ------^ U G ---- A**

**zma-miRn62c UGGACAAGAUUUGAUGUUAGC**

**ACGA G CUA C .-AG| AG**

**UUAG AUGCUAACAUCAAAUUUUGUCUAUUGGAUUCA UCUAUGG UGAGUGCAAUUCCACC GGGAU \**

**GAUU UGcgauuguaguuuagaacagguAGCCUAGGU AGAUACC ACUCACGUUAGGGUGG UCCUG C**

**CAC- G AGG A \ --^ UG**

**zma-miRn62d** **UGGACAAGAUUUGAUGUUAGC**

**UAUUA A U C C CCA C C .-C| AC**

**GGCGUGCUAACAU AAAUCUU UCCA UAGAUC ACCAUCUG CUG GUGUAAUC CA UAGGG G**

**UUGUAcgauugua uuuagaa aggu AUCUAG UGGUAGAU GAU CACGUUAG GU AUCCU A**

**UACGA g c U A ACA U A \ -^ AC**

**zma-miRn63 GCAGCCGUCGGACAUAACGG**

**UCCGACGG C UA-| AGA A**

**CUCCGUUAUGUCCG CGGCUGCU GG CCGUCGGACAUA G**

**GAggcaauacaggc gccgacgG CC GGCAGCCUGUAU C**

**UCCUAUAA u UCG^ --- U**

**zma-miRn64 UCUGUUUGAAUGCACUAGAGC**

**- CUU g - C .-U .-CAUCCAAACAA| UU**

**GUA AGGGucuguuugaaugcacua agcUAAUAGUUAGCU GCU AAA UAGCUG--GAGA CCAGCUAAUAG \**

**UAU UCCCGGACAAACUUACGUGAU UUGAUUAUCAAUCGA CGA UUU AUCGAC CUCU GGUCGAUUAUC C**

**A AAU G C U \ - \ \ -----------^ GA**

**zma-miRn65 AAUACACAUGGGUUGAGGAGG**

**- a -| A**

**UAUUGACCCCaauacacauggguugagg ggAUUG GGUGUAAAUUA \**

**AUAACUGGGGUUAUGUGUACUCAACUCC CCUAAC CUACAUUUAAU A**

**C A U^ C**

**zma-miRn66 AGACUGCCAGAAUUUUCGUGCA**

**UUGUG - -| uuuu UC A**

**CU GUCagacugc cagaa cgugcaG AAC \**

**GA CAGUUUGACG GUCUU GCGCGUC UUG C**

**CCAAA C C^ UUUC UU C**

**zma-miRn67 UAAUGGAUCAAAUGGUUUGCU**

**G A C A AC .-ACAGA| A UG UUAG U**

**CAA UGUGCAAGCAAAUCAU UGAUUCAUUA AUU GAUUCAACC CAC AUCA GUUUG GGUUUUU U**

**GUU AUACGUucguuuggua acuagguaau UAG CUAGGUUGG GUG UGGU CGAGU UUAGAAA A**

**- C a C CA \ -----^ G -- UA-- U**

**zma-miRn68** **UCUCCAUGGGCUGCGUCGCAU**

**A| GCCAGG UG .-GAAGCCAUGAGAUC AUC**

**CGAAGGUGGCAUGUGGCGCAGUCCAUGGAGAUGGGGGCGCCGGUU ACGACGCCG AAAUUGGCAGCA GG A**

**GCUUCCACCGuacgcugcgucggguaccucuACCCCUGCGGUCAA UGCUGCGGC UUUAACCGUCGU CC A**

**-^ ------ GU \ -------------- CAA**

**zma-miRn69** **CCCUAAGGGUUUGUUCGGUUAU**

**G UGAA- -----------| G A AA CA A**

**GAU AAUAACCGAACAAGCCCUUAG UG UAAGGGAUCCCAAGGGGAUUCAUGGG GA AAUCCCCUUG AUUCA U**

**CUA Uuauuggcuuguuugggaauc AC AUUCCCUAGGGUUCCCCUAGGUACCC CU UUAGGGGAAC UAAGU U**

**A UAUCC ccAACUAAACU^ G - CC AA U**

**zma-miRn70** **AAGUUACAGAUGUAUGGAGCA**

**CUUA A | UC**

**UAAAAUUGCUCCAUACA CUGUAACUUUGGACUAAAC--UC C**

**GUUUUAacgagguaugu gacauugaaACCUGAUUUG AG U**

**AAAC a \ ^ UU**

**zma-miRn71** **UUAGGCUCGGGGACUACGGUG**

**C A cu ga C UG .-CUAU| A A**

**GGCCU ACAuuagg cggg cuacggug AGCCAAGGA ACUUGCCGAU CGUCG UC \**

**UCGGA UGUAGUCC GUCC GGUGCCAC UCGGUUCUU UGAACGGCUA GCAGC AG A**

**U A UC UC A GU \ ----^ G C**

**zma-miRn72** **UGAACCGGUUUUCUGUUUUC**

**--| UG C UU -- A**

**AGUU ACCAGAAA CGG GAACCGGUUU CUG \**

**UCGG UGGUcuuu guc uuuggccaag GAC A**

**AC^ -- u u- UU C**

**zma-miRn73** **UUGAACCGGUUUUCUGUUUUC**

**UUA| A aa AA U AGG**

**GAGU GAuug ggggauuggagaggauUAAAUCCCCUCUUAUAC AUUG AU A**

**CUCA CUAAC CCCCUAACCUCUCCUAAUUUAGGGGAGGAUAUG UAAU UA G**

**AAC^ C -- C- U GGG**

**zma-miRn74a** **UAACUAGCCGUCGGAGAUAAG**

**GUUAAAAAA| U UAA**

**GCUUAUCUCCGACGGCUA UUACGAGGCCGUCGGAUA C**

**Ugaauagaggcugccgau aauGCUCCGGCAGCCUGU C**

**CUUUAUAUC^ c UUU**

**zma-miRn74b** **UAACUAGCCGUCGGAGAUAAG**

**GUUAAAAAA --| A**

**GCUUA UCCGACGGCUAGUUACGAGGCUGUCAGAUAUA C**

**Ugaau aggcugccgaucaaUGCUCCGGCAGUUUGUAU C**

**CUUUAUAUC ag^ U**

**zma-miRn75** **UCGCUAGAUCGUUGAGGGAUU**

**A - C A .-A C--| AUCC**

**AUUCC CUCCAAUCCCUCA CGAUCUAGC ACCAUAACAGU GGCUACAU UAUG A**

**UGGGG GAGGuuagggagu gcuagaucg uGGUAUUGUCA CCGAUGUA AUAC U**

**- A u c \ - CGU^ CACA**

**zma-miRn76** **CAAAGAAAGUAGCCACAUGCCU**

**A| C G ACA A- UAAGAU**

**UGA AUCACAAGGCAUGUGGC UGC CU GUUA \**

**ACU UAGUGUuccguacaccg aug ga cAAU A**

**-^ C - aaa aa UAUGGU**

**zma-miRn77 F1-m1067-3p UCUAGAUCCAACGGACCAAAA**

**F1-m1067-5p UUGGUCCGUUAGAUCUAGAUC**

**G C a A | U**

**CAAG AGCUCUUuugguccguu gaucuagaucUAA CGUAU--GCUA A**

**GUUU UCGAGaaaaccaggcaa cuagaucuAGGUU GCAUA CGAU U**

**- A c G \ ^ U**
